# Opportunities and challenges in training volunteer wildlife detection dog-handler teams for invasive plant monitoring

**DOI:** 10.1371/journal.pone.0359003

**Published:** 2026-11-04

**Authors:** Annegret Grimm-Seyfarth, Wiebke Harms, Veronika Paulina Koch, Nora Haack, Alexander Harpke, Katja Krauss, Anne Berger

**Affiliations:** 1 Department of Conservation Biology and Social-ecological Systems, Helmholtz-Centre for Environmental Research – UFZ, Leipzig, Germany; 2 Independent Institute for Environmental Issues, Halle, Germany; 3 Society for the Resocialisation and Training of Dogs, Berlin, Germany; 4 Department of Evolutionary Ecology, Leibniz Institute for Zoo and Wildlife Research, Berlin, Germany; University of Bucharest, ROMANIA

## Abstract

Measures to combat invasive alien species (IAS) are costly and time-consuming, making effective and widely available detection methods essential. Wildlife detection dogs (WDD) are effectively used to detect hidden and cryptic species, offering enormous potential for early detection of IAS. While professional WDDs are rare, private dog owners are looking for meaningful activities. Therefore, we combined online dog training, practical seminars and field tests to train 31 companion dogs and their owners as volunteer WDD teams. We selected three IAS, the *Fallopia* spp. complex, *Impatiens glandulifera* and *Ambrosia artemisiifolia*, as target species due to their prevalence and strong impacts on biodiversity, economy and health. We used detailed video documentation and questionnaires to investigate the effects of inherent (social) parameters, training procedures and environmental conditions on performance and stamina of individual dog-handler teams during training in two cohorts. In total, 87.1% of the participants completed online training, 77.4% the practical seminar and 35.5% conducted the final field test. Both cohorts were comparably fast and accurate during online training, but cohort 1 performed better in the practical seminar. Performance during online training was mostly associated with social factors and reward types. In contrast, best results in the practical seminar were achieved for larger plant parts, when the participants had included all plant parts in their previous training and were able to adapt the training plan slightly. Spontaneous generalisation rate from thawed to fresh laid-out plant parts was 56%. Of the 12 teams conducting the field test, 11 teams passed. Neither the performance during online training nor during the practical seminar ultimately affected operational capability, but personal motivations determined whether a team finished the program. Finally, volunteer WDD teams can demonstrate high efficiency in monitoring, but the intensive training must be carefully monitored by professional ecologists and dog trainers.

## Introduction

Invasive alien species (IAS) are characterised by their ability to colonise new locations very quickly, multiply en masse and impair naturally occurring ecosystems, biotopes or species [1], which is why their intentional and unintentional spread outside their natural range is one of the main causes of biodiversity loss worldwide [2]. As they pose an increasing ecological and economic problem [2, 3], effective management requires their early detection and control. One approach that has seen limited use in Europe for monitoring species, including IAS, is the use of detection dogs. Unlike humans, dogs can distinguish the odour of individual animal and plant species down to the level of individuals [4], track down the target species in a shorter time [5] and also locate individual scents or trails with high accuracy and from a greater distance [6]. The success of using detection dogs to detect species when monitoring a wide range of wild animal and plant species has already been proven in many studies, also in comparison with other detection methods (e.g., [6–10]). Wildlife detection dogs (WDD) are not only more effective, but often also more economical to determine the presence and abundance of hidden plant or animal species in a non-invasive way [6, 10, 11]. Accordingly, WDD are particularly useful when the target species are inconspicuous, obscured by other materials (like soil) or visually difficult to perceive [12], which is the case for different developmental stages of plants, such as roots, stem parts or seeds. This is especially important for the Early Detection and Rapid Response (EDRR) management of invasive species, as species in the spreading-phase of the invasion show low abundances and thus tend to be inconspicuous. Dogs can be trained to find different parts of a plant species, including stolons, roots and rhizomes, even if they have previously been trained with a specific plant part only [13–15]. Through the process of generalisation, dogs develop so-called ‘search images’ during training, shaped by their experiences during previous searches and finds [16]. In contrast, the visual appearance of a sample does not affect the success to distinguish odours in the olfactory search image [17]. Thus, dogs exhibit a much lower reliance on visual cues and knowledge-based location when searching for their targets, ignoring non-targets at the same time, whereas humans often restrict their search to locations previously associated with the species’ presumed habitat [10, 18]. Together, this enables detection dogs to reveal new aspects even for a supposedly well-researched species and also detecting early reproductive stages, which is crucial for early detection of colonisation and shortens the time required for the local eradication of IAS.

In addition to professional dog handlers, first studies have shown that it is possible to train private dog owners and their pet dogs as volunteer WDD teams to support nature conservation and detect certain species, if a good basic professional training is provided [19]. Measured by the number of participants and the public awareness of the dogs, this can also sensitise a large proportion of the public to the topic of IAS [13]. Since the use of volunteers in nature conservation has been established for many decades, a pilot project called IGAMon-Dog was launched to recruit volunteer human-dog teams to search for three selected neophytes in Germany. The three neophyte species used in this study were selected based on their effects on economics, human health and biodiversity as well as a broad distribution range in Germany. We combined online dog training, practical seminars and field tests to train 31 companion dogs and their owners as volunteer WDD teams. Training was regularly evaluated and assessed using online seminars and video assessments. In addition, questionnaires were used to examine further social, training and motivational factors.

The overall aim of this study was twofold. First, we evaluated the feasibility of training volunteer dog-handler teams to detect invasive plants and to progress successfully through a structured training programme to field deployment. Second, we identified social, training, and environmental factors associated with successful and efficient training in order to derive recommendations for the selection of teams and the design of future volunteer WDD training programmes. To address these objectives, we analysed the successive phases of the training programme separately. For the online dog training, we determined (Q1) which social and training factors influenced how quickly training goals were achieved, and (Q2) whether the goal was correctly achieved. For the practical seminar, we determined (Q3) which social, training and environmental factors influenced the detection of plants and (Q4) overall team performance. For the final field test, we determined (Q5) whether performance during online training and the practical seminar as well as the type of motivation of the handler for conducting the programme influenced whether a team conducted the field test. Overall, we hypothesised that (1) volunteer teams would show substantial variation in training success and performance throughout the programme; (2) higher training effort (longer, more complex and diverse training) would be associated with improved performance; (3) successful completion of one phase of the training programme would be associated with a higher likelihood of successful performance in subsequent phases.

## Materials and methods

### Target plants

The three selected IAS – the *Fallopia* spp. complex, *Impatiens glandulifera* and *Ambrosia artemisiifolia* – were chosen for their significant impacts on economics, human health and biodiversity as well as their widespread distribution in Germany. This broad occurrence ensured that volunteer WDD teams that chose their target plant themselves had the opportunity to find it in their local area. The knotweeds (*Fallopia* spp.) are among the IAS with the highest potential impacts [20]. The complex includes the Japanese knotweed: *F. japonica*; the giant knotweed: *F. sachalinensis* and their hybrid, the Bohemian knotweed: *F.* × *bohemica* [21]. They were brought to Europe for horticultural use in 1848 and are widespread by now. Of the three variants, the Bohemian knotweed is the most competitive and therefore more detrimental to native plants [21]. The *F*. spp. complex is classified as IAS for their detrimental effect on a variety of native species, the extent to which they alter soil biochemistry and their resulting socio-economic impacts. They can restrict accessibility and visibility of infrastructure such as railways, waterways or roads, cause damage to structures such as dams and promote erosion of the riverbed [21–23]. Critical stages for EDRR include small parts of stolons and rhizozoms, where the plant can regrow, as well as seedlings ahead of larger stands.

The Himalayan balsam (*I. glandulifera*) is an IAS that exhibits differential effects on native flora and fauna. While some species, such as shade-tolerant plants or certain invertebrate herbivores, may benefit from the presence of *I. glandulifera*, other species are significantly negatively impacted [24–26]. Additionally, it can contribute to riverbank erosion and cause eutrophication of water bodies due to the autumnal dieback of large stands [26]. Overall, although *I. glandulifera* is a widespread IAS, it is less likely to negatively affect native flora and fauna compared to the *Fallopia* species. Critical stages for EDRR are mainly seedlings, which can be found far away from larger stands as seeds are shot far away and can also be transported by rivers, along which they mainly occur.

The common ragweed (*A. artemisiifolia*), native to North America, is also listed as an IAS and is highly allergenic [27]. Furthermore, it can reduce species richness and the pastoral value of affected areas [28]. Studies suggest that the impact of *A. artemisiifolia* on human health, and consequently on the economy, could result in costs of up to €7.4 billion annually in Europe [27]. As plants occur as single individuals, each individual found would contribute to EDRR.

### Volunteer WDD team selection and general training programme

A total of 30 volunteers with their dogs (31 human-dog-teams, as one volunteer worked two dogs in total) were selected for participation in the project, divided into two cohorts of 15 and 16 teams from a total of six German federal states (S1 File). Selection of volunteer WDD teams was based on previous experience of the handler with dog training, sport or scent work (in ascending ranking), the statement about the motivation to contribute to this project (where experience in conservation, plants or invasive species was giving a higher rank), available time budget (the more the higher ranked), training programmes or examinations of the dog, health or anxiety statements about the dog (severe issues led to exclusion), and dog age, where only dogs between 12 months and 10 years were taken for dog health reasons. Dog breeds were classified according to the Fédération Cynologique Internationale [29]. Dog owners provided written informed consent during the application process, which was subsequently reaffirmed verbally following acceptance into the project.

The detection dog training of the first and the second cohort started in September 2021 and September 2022, respectively. Training followed a backward structure [30], was comparable among cohorts and divided into three phases (Fig 1, detailed descriptions below). Phases 1 (learning a passive alert) and 2 (learning a structural search) comprised an online dog training for around six months, accompanied by video-assessments. During this time, two experienced detection dog trainers provided a detailed training plan per phase for the participants. Monthly online seminars allowed for monitoring individual progress and explaining the theoretical background for the upcoming steps. In addition, the participants had the opportunity to show the video recordings to the trainers via the project’s internal forum, who then provided feedback on any difficulties and tips for further training based on the recordings. Phase 1 was finished with the dog indicating on target odour at short distance with a five seconds stare alert through video assessments. Phase 2 finished with a practical seminar, which was organised with a third experienced detection dog trainer on site, with 5–6 participants per day. At these seminars, the current training status was evaluated and an individual plan for further training on the participants’ own initiative was drawn up. During the practical seminar, participants were asked to fill a questionnaire about their training (S2 File). Training then continued with phase 3 (wild target search) on a self-organised basis with independent local training and the option to contact all three trainers one-by-one. At the same time, the participants were given tutorial online seminars to prepare them for surveying their target species during field work. Phase 3 was finalised shortly before the field deployments through a field test, which reflected the operational capability of the team. After one year, each team was asked to evaluate the project by means of a professional evaluation form, created using a combination of the scale system Motives and Framework for Volunteering in Citizen Science (MORFEN-CS [31, 32] and modifications suggested by [33], both of which are based on the Volunteer Functions Inventory (VFI [34],). While we will not explain the whole evaluation process here, we will include the handler’s motivation to complete the programme (S3 Table) in some analyses.

**Fig 1:**
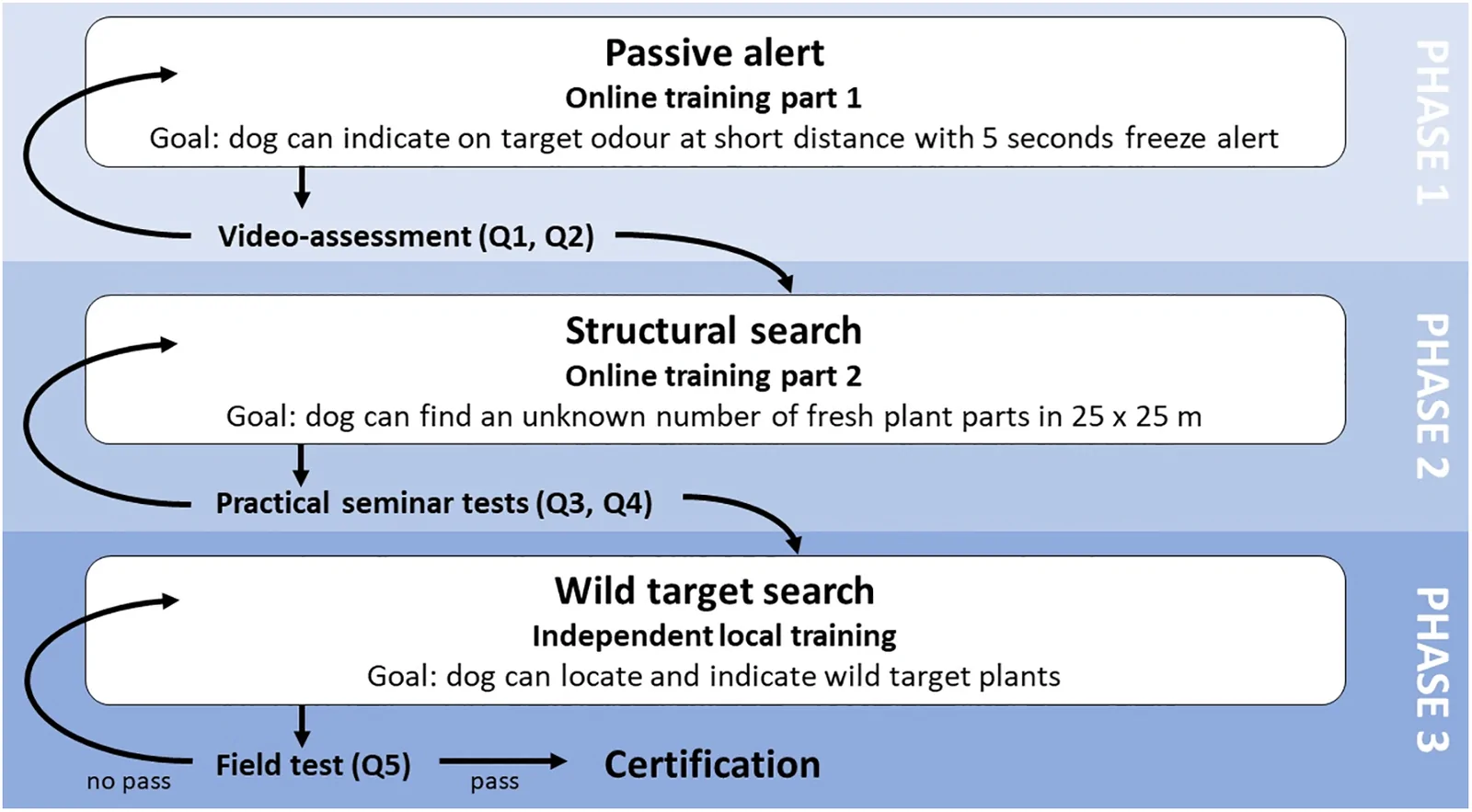
Overview on the three training phases. Phases 1 and 2 were conducted via online dog training and video assessments, Phase 2 was completed via a practical on-site seminar, and Phase 3 was conducted independently (with optional individual consultations with dog trainers) and completed with a field test. The question numbers are added behind the assessments and refer to the phase analysed to answer the respective question.

According to the German Animal Welfare Act (Tierschutzgesetz, § 7 ff.), the procedures conducted in this study did not constitute an animal experiment and therefore did not require approval by an animal research ethics committee or a formal ethics permit. The study design and procedures were discussed with the institutional Animal Welfare Committee of the Helmholtz-Centre for Environmental Research, which confirmed that no formal ethical approval or permit was required. All dogs were trained exclusively by their owners, under the guidance of certified dog trainers licensed by the local veterinary authority; no dog was trained or handled by the researchers. All training and testing procedures relied solely on positive reinforcement, without coercion or punishment, and were conducted in compliance with applicable animal welfare regulations. The welfare of the dogs was ensured at all times, and no animals were harmed during the study. The dog owners provided written informed consent to participate in the project, and no identifiable information was collected from the human participants.

### Online dog training

The online dog training was divided into two phases, where phase 1 aimed at training the dog with a passive alert and phase 2 was training a structural search (Fig 1). All participants were given a detailed training plan per phase and met once a month for an online training seminar, where live exercises were shown and practised in front of the camera. The individual training progress was documented by the participants on video and posted weekly in a forum, where they received feedback from the dog trainers in order to adapt the training to their own individual level of performance. Videos sent to the trainers were also used to check whether single training steps of the training plan had been successfully completed. If necessary, participants were asked to repeat previous steps before moving on, but the implementation of the repetition was not checked to minimise performance pressure on the voluntary participants. A summary of the training steps is given in Table 1.

**Table 1:** Description of the main training steps in online training parts 1 and 2 (the complete detailed plan contained 16 steps in part 1 and 10 steps in part 2). Sample odour in part 1 means KONG(R) (or similar hard rubber toy) for cohort 1 and target scent (plant parts) for cohort 2. Detailed steps are the number of detailed steps in the training plan to reach the main training step. An “x” in columns cohort 1 and 2 symbolises which steps were applied.

Part	Training step	Training goal	Number of detailed steps	Cohort 1	Cohort 2
1	1	Sample odour is held slightly above height of dog’s head; when the hand stops, dog looks at it with 10–30 cm distance for at least 5 seconds	3	x	x
1	2	Marking line on the floor between dog and sample odour (to keep distance), otherwise as step 1, 5 seconds alert	2	x	
1	3	Sample odour lies on the floor (marking line removed), command introduced, 5 seconds alert	5	x	x
1	4	Sample odour on the floor next to the owner, 0.5 m distance to the dog, upon command dog goes to sample odour, 5 seconds alert	2	x	x
1	5	Sample odour is downsized (stepwise), 5 seconds alert	1	x	x
1	6	Dog is sent to the sample odour, sample odour at a distance on the floor (stepwise up to 3 m), 5 seconds alert	2	x	x
1	7	Changed locations, distractions added, otherwise as step 6	1	x	x
2	8	Target scent and KONG presented together, 1 second alert	3	x	
2	9	Target scent presented only, 1 second alert	1	x	
2	10	Introduction of a new command for the target scent, 5 seconds alert, otherwise as step 9	2	x	
2	11	Target scent hidden in various places and invisible to the dog, but dog can watch while hiding, dog sent to search on command, 5 seconds alert	2	x	x
2	12	Introducing more distractions, dog should not watch while hiding, increasing number of single- and double-blind searches, dog sent to search on command, 5 seconds alert	2	x	x

Phase 1 of the training plan included steps teaching the dogs to give a passive alert, i.e., to stand/sit/lay (dog-specific) and stare at a substitute or target odour [30]. The aim of a successful passive alert is that the odour sample is not touched by the dog and the dog freezes until a response is received from the handler. The specific alert posture (sit-, lay- or freeze-and-stare) was left to the discretion of each team, provided it included a clearly defined pause. This pause later serves as a reliable and unambiguous indication of scent detection during field searches. The duration of the alert has been gradually increased and distractions (e.g., movements by handler, other pets, toy or food on the ground) introduced [30, 35]. Cohort 1 began training with a substitute odour – a hard rubber toy, often a KONG(R) by the KONG company – which was gradually reduced in size over the course of the training. This object was eventually conditioned to the target odour at the beginning of phase 2 (Table 1). Cohort 2 trained right from the start with their target odour. The target odour per team included various dried or thawed plant parts frozen between training sessions of one of the three target plants, selected for the team by the coordinators depending on occurrence in proximity to the team’s home and, if several plants occurred, personal preferences. Another difference was that training step 2 in cohort 1 included a marking line on the floor between dog and the substitute odour, which was removed in cohort 2. These adjustments were made in response to feedback from cohort 1 participants, who assessed the step time-consuming and of limited practical value. Importantly, the study was not specifically designed to test the different approaches applied to each cohort. Rather, minor opportunistic modifications were made between cohorts that will be accounted for statistically. Apart from that, training steps were identical for both cohorts (Table 1). Phase 1 was considered completed when the dog was able to indicate on the substitute or target odour at short distance with an at least 5 seconds freeze alert (Fig 1, Table 1).

After the training goals for phase 1 (passive alert) were reached, phase 2 started (Fig 1). To keep motivation as high as possible, participants were asked to determine the start of phase 2 individually or together with a trainer instead of taking an intermediate exam. The distracting stimuli were reduced again and the odour sample was slightly hidden in order to teach the dogs the first steps of a search, which ended with an indication in front of the odour. The training for a successful search was also built up stepwise and the degree of difficulty and distraction was increased. The final training step involved the dog locating and indicating the target odour sample at an unfamiliar location (Table 1). Phase 2 was completed by the evaluation during the practical seminar (Fig 1, see below).

The entire training concept was assessed (Q1 and Q2) on the basis of the videos provided by the participants. A training step was analysed when at least five participants provided videos for it. Since this was not the case for every single training step, the detailed training plan was clustered to 12 main training steps (Table 1).

Various criteria were selected for analysing the training videos of the online training parts 1 and 2 to ensure that all relevant aspects of the training process were captured (Table 2). We focused on the dog’s behaviour concerning the alert at the substitute or target scent. As there were occasionally several videos per participant for one training step, we analysed the first video in which the training goal for this step was achieved and ignored all subsequent videos for this step. We determined whether a team had achieved the goal of a training step on the basis of the correct indication (true positive) and necessary alert duration (see Table 1). An alert was considered interrupted if the dog clearly moved its head or changed its position. After each video sequence viewed, the following criteria were noted: the team ID, how many days the team needed to achieve the goal (counted as days since the first online tutorial), whether the goal was truly achieved, whether the reinforcement was given correctly, the total duration of the alert, the indication category, and potential distractions (see Table 2 for details). The previously defined criteria (referred to as ‘video parameters’ in the following) were then linked to related background information and inherent factors of the teams (referred to as ‘social factors’ in the following) (Table 2). For each training step, we calculated the average date when a training goal was first achieved across all teams per cohort. We then calculated the deviation from average duration as the difference between the date on which each single team reached the training goal and the cohort-wide average date in days, where positive deviation indicates that the team reached the goal later than average and negative deviation indicates that the team reached the goal earlier than average (Table 2). This metric enables the comparison of individual team performance relative to cohort norms, accounting for variability in training pace. Furthermore, for each team, we calculated the proportion of successful training steps (i.e., the goal was achieved) in relation to the number of filmed training steps (as often teams did not provide videos for all main training steps) for the online training parts 1 and 2. Thus, while we only considered videos from teams that reached the goal of a particular training step in calculating the training pace, we used all filmed training steps to calculate the relative proportion of successful training steps.

**Table 2:** Parameters per team examined during the study grouped by data collection method, the parameter class and the parameter definitions. Social factors were examined by a questionnaire at the beginning of each cohort and confirmed during the practical seminar; Video parameters were taken during video assessments; On-site conditions were measured and taken during on-site test sessions at the practical seminar; Training parameters were requested through a questionnaire during the practical seminar; Performances are the measures taken to assess team performance in a particular training section; and Motivation was assessed through the evaluation after one year (see S3 Table for longer definitions). (C) behind parameters indicate controlled variables experimentally manipulated or based on design, (O) behind parameters indicate observed variables recorded as they naturally occurred. Relevant questions where parameters were considered as explanatory variables are given in the last column (see statistical analyses section for details), whereby parameters which are response variables for a particular question are highlighted in bold.

Group	Parameter	Class	Definition	Question
**Social factors**	Cohort (C)	categorical	Cohort 1 (C1): Start September 2021, practical seminar in 2022; Cohort 2 (C2): Start September 2022, practical seminar in 2023	Q1-Q5
	Dog age (O)	integer	Age of the dog at the beginning in years	Q1, Q2
	Dog sex (O)	categorical	Sex of the dog: male, female	Q1, Q2
	FCI (O)	categorical	Breed according to FCI groups (1 = Sheepdogs and Cattledogs, 2 = Pinscher and Schnauzer, 3 = Terriers, 4 = Dachshunds, 5 = Spitz and primitive types, 6 = Scent hounds and related breeds, 7 = Pointing Dogs, 8 = Retrievers, Flushing Dogs, Water Dogs, 9 = Companion and Toy Dogs, 10 = Sighthounds) (*FCI Breeds Nomenclature*, 2024). Mixed breeds were assigned an additional group 0.	Q1-Q4
	Target species (C)	categorical	Target species the dog was trained on: *F.* spp. complex, *A. artemisiifolia* or *I. glandulifera*	Q1-Q4
	Team (C)	categorical	Team ID (a number created by the computer system)	Q1, Q2
	Team experience (O)	categorical	Based on S2 File answers for the dog: 0 = none, 1 = dog sports, 2 = tracking and general scent work, 3 = scent matching or object search	Q1-Q4
**Video parameters**	Alert duration (O)	integer	The total duration of the alert in seconds (could be longer than necessary for this step)	Q1, Q2
	Chosen alert behaviour (O)	categorical	Alert trained: freeze, sit, or lay and stare at target	Q1-Q4
	Distraction (O)	integer	Number of distractions seen in the video, e.g., other dogs, traffic, wildlife; between 0 and 3	Q1, Q2
	Indication category (O)	categorical	Based on signal recognition theory [36]: true positive: the dog correctly indicated the sample odour; true negative: the dog did not indicate because there was no sample odour (empty search); false positive: the dog indicated although there was no sample odour; or false negative: the dog did not alert although the sample odour was present.	Q1, Q2
	Minimum alert duration (C)	integer	The required alert duration in seconds according to Table 1	Q2
	Reinforcement (O)	binomial	Reinforcement was given correctly (1) or not (0); Reward immediately after the dog’s correct alert was considered correct; if the reward was given too late or after the dog’s incorrect behaviour, the reward was assessed as incorrect	Q1, Q2
	Reward type (O)	categorical	Reward type used: food, toy, or both	Q1-Q4
	Sample odour (C)	categorical	Only relevant for cohort 1: whether a training step was performed on Kong or plant	Q1, Q2
	Training step (C)	categorical	Training step according to Table 1	Q1, Q2
**On-site conditions**	Grid (C)	integer	Grid where the specific plant sample was hidden	Q3
	Percentage of fresh samples (C)	numerical	Percentage of fresh samples (vs. thawed) in all test sessions per team (only plants considered that the dog had passed), between 0 and 1	Q4
	Sample condition (C)	categorical	Condition of the hidden sample in the test (fresh or thawed)	Q3
	Sample length (C)	integer	Length of the laid-out sample in cm	Q3
	Temperature (O)	numerical	Temperature in °C	Q3, Q4
	Test session (C)	integer	Test session number (see text for details)	Q3
	Time (O)	numerical	Time of the Test session	Q3
	Weather (O)	categorical	Weather conditions: sunny, partly cloudy, cloudy, rainy	Q3, Q4
	Wind (O)	categorical	Wind strength checked by observing trees following [37]; none, slight, intermittent, moderate, or severe, referring to Beaufort scale 0–1, 2, 3, 4, 5 or more, respectively	Q3, Q4
	Wind direction (O)	categorial	Direction where the wind came from	Q3, Q4
**Training parameters**	Dried (O)	binary	Were dried plants included in training? (1 = yes, 0 = no)	Q3, Q4
	Fresh_training (O)	binary	Were fresh plants included in training? (1 = yes, 0 = no)	Q3, Q4
	Participation online training seminar live (O)	categorical	Live participation in the online training seminars (yes, partly, no)	Q3, Q4
	Plant parts (O)	integer	Which/how many parts of the plant were used for training (1 = leaves, 2 = leaves and stem, 3 = leaves, stem and roots, 4 = leaves, stem, roots and flower)	Q3, Q4
	Search area (O)	numerical	Average size of the search area of a training session in m²	Q3, Q4
	Session duration (O)	numerical	Average length of a training session in minutes	Q3, Q4
	Single blind (O)	categorical	Number of single blind searches (none, few, many)	Q3, Q4
	Steps skipped (O)	categorical	Number of skipped steps in the detailed training plan (none, few, many)	Q3, Q4
	Training days/week (O)	numerical	Average number of training days per week a team could afford	Q3, Q4
**Performance**	De.ave.duration (O)	integer	Training pace or deviation from average duration as the difference between the date on which each single team reached the training goal and the cohort-wide average date in days, scaled per cohort; see text for details	**Q1**, Q2
	Diff_goal (O)	numerical	Mean of deviation from average duration to reach a training goal (goal achieved = yes) in phase 1 and 2 (online training), i.e., mean of *De.ave.duration* across all training steps, in days	Q3-Q5
	Goal achievement (O)	binary	Whether a training goal during online training was achieved in a specific training step (1) or not (0), separate per training step	**Q2**
	Rel_goal (O)	numerical	Relative goal achievement, a normalized metric that quantifies the proportion of training steps in which a specific training goal was successfully achieved, relative to the total number of training steps filmed per team during phase 1 and 2 of online training; 0 indicates the goal was never achieved across any filmed step, 1 indicates the goal was achieved in every filmed step, values between 0 and 1 reflect partial achievement relative to the total available training steps filmed	Q3-Q5
	Detection (O)	binary	Whether a dog detected a target sample they had passed in the test sessions of the practical seminar (1) or not (0), i.e., separate per plant hidden (only plants considered that the dog had passed)	**Q3**
	Proportion detected (O)	numerical	Proportion of plants successfully detected by the dog during the practical seminar, relative to the total number of plants that the dog had passed during the session (i.e., plants that were within the dog’s operational range and were potentially detectable); value ranges from 0 to 1, where 0 indicates no detection among all passed plants, 1 indicates detection of all passed plants, and values between 0 and 1 reflect partial detection performance	Q5
	Team score (O)	numerical	Teams received points for starting and finishing test sessions, points were multiplied by the proportion detected (see text for detailed calculation) in practical seminar, between 0 and 9	**Q4,** Q5
	Field test conducted (O)	binary	Whether a team has conducted the field test (1) or not (0), independent of the performance during the field test	**Q5**
**Motivation**	Career (O)	integer	Evaluated by handler, between 1 and 6, see S3 Table	Q5
	Community (O)	integer	Evaluated by handler, between 1 and 6, see S3 Table	Q5
	Conservation (O)	integer	Evaluated by handler, between 1 and 6, see S3 Table	Q5
	Dog training (O)	integer	Evaluated by handler, between 1 and 6, see S3 Table	Q5
	Qualification (O)	integer	Evaluated by handler, between 1 and 6, see S3 Table	Q5
	Responsibility (O)	integer	Evaluated by handler, between 1 and 6, see S3 Table	Q5
	Science (O)	integer	Evaluated by handler, between 1 and 6, see S3 Table	Q5
	Self-efficacy (O)	integer	Evaluated by handler, between 1 and 6, see S3 Table	Q5
	Work-life balance (O)	integer	Evaluated by handler, between 1 and 6, see S3 Table	Q5

### Practical seminar tests and questionnaire

The practical seminar tests were scheduled at the end of the online training phase as on-site training. A total of 24 teams (11 from cohort 1 and 13 from cohort 2) were able to participate. All teams were checked for their current training status in three test sessions and then individually supervised by an additional experienced detection dog trainer and the project staff. In parallel, participants were asked about their training habits at home using a questionnaire (S2 File). We categorised the answers into ‘training parameters’ (Table 2). During the test sessions, the dogs were also presented with fresh odour samples (grown and harvested as young plants) of their target species. For those who had not previously trained with fresh plants, we determined whether the different dogs were able to generalise from thawed or frozen to fresh plants. In each of the three test sessions, either one or two samples of the target species had to be found. The teams had to search a predefined short grass area of 15 x 15 m (session 1 and 2) or 25 x 25 m (session 3) in a maximum of 5 or 10 minutes, respectively. Search areas were divided into 5 x 5 m grid cells (i.e., 9 in sessions 1 and 2, 25 in session 3) to simplify preparation. A random number generator was used to determine the number of samples (1 or 2) and location within the grid for each team and session, whereby only one sample per grid cell was allowed (pulling without replacement). Based on this protocol, all three sessions had been prepared by the coordinators before a team started. This person was unfamiliar to the dog, walked through all grids in all areas and touched the ground several times without hiding something to reduce the likelihood that a dog will track the person. Samples were left for at least 15 minutes prior to start. Neither the teams nor the trainer knew the number of samples hidden per session or their position, only the coordinators did (single-blind tests). A session was completed when the team either found two odour samples, or found one odour sample and said that no further sample was hidden, or the maximum time had expired. The team could also cancel the search for personal reasons, resulting in an incomplete session. The team could also decide whether the dog would manage to do all three test sessions or only one or two of them. If the dog appeared stressed or fatigued to the trainer, she would have stopped the team as well. For each test session, additional parameters (‘on-site conditions’) on weather conditions and sample properties were recorded by the coordinators (Table 2). We hereby distinguish between variables that were experimentally controlled or design-based and those that were observed only to ensure that causal claims are grounded in the experimental control, while correlative findings are appropriately interpreted as associations rather than effects.

In order to better assess the general performance of the teams (Q3 and Q4) and to compensate for incomplete test rounds or variable levels of performance, a score system was developed. Each team received one point for each test session started (three sessions in total: max. 3 points). An additional point was awarded for completing the first session, two points for completing the second session and three points for the third session (max. 6 points). This difference was chosen because the complexity increased between test sessions, with introducing fresh plant samples in session 2 and increasing the area in session 3. The team score was then calculated by multiplying the total number of points with the percentage of hidden samples found successfully (proportion detected of those samples passed by the team) to calculate the overall success rate. Teams could achieve a maximum score of 9 when all test rounds were started, finished and all hidden samples detected.

### Field test

Before the teams conducted their own surveys of their target species in the field, they completed a field test, which they could carry out independently with a helper or with one of the coordinators. The test consisted of two searches in the real habitat of the target plant. The first search was carried out on a 25 x 25 m area or a 200 m long, 3–5 m wide transect, where up to 5 (preferably) fresh samples of the target plant were hidden by the helper or coordinator who was not present during the search, so that the teams did not know the exact number and position of the samples beforehand or could be confirmed directly (double-blind search). The second search was designed to demonstrate the team’s ability to find and alert on wild plants in an area where their presence was generally known. Both searches were filmed and sent to one of the dog trainers for evaluation together with the protocol sheet completed by the dog handlers on weather conditions, sample characteristics and the dog’s alert behaviour (S4 File). A team passed the field test when it could at least successfully find 50% of the hidden samples in a double-blind search, based on the reports of [38]. Due to individual differences in the implementation and the plants (parts) used during the field test, we later only analysed whether a team completed the field test at all or not (Q5).

### Statistical analyses of team performances

We performed separate analyses per research question Q1-Q5 representing the different training sections (Fig 1, Table 3) using (generalised) linear models [(G)LM] [39]. For the online dog training, we determined (Q1) which factors influenced how quickly the goal of a training step was achieved, using the deviation from average date to reach a training goal (negative: faster than average, positive: slower than average) as a response variable with Gaussian error distribution. We only included teams that reached the training goal in the respective training step. Due to slight differences in the training plan between cohorts affecting the timing of training steps, we scaled the response per cohort to a mean of 0 and a standard deviation of 1, to keep the average per cohort before combining data. We further determined (Q2) which factors influenced whether the goal of this training step was achieved, using a binomial error distribution (1 = the goal was achieved, 0 = it was not). In both analyses, explanatory parameters comprised all social and video parameters (Table 2). As these were too many parameters to be analysed together (overfitting), we divided the two (G)LMs into three sub-models each for specific hypotheses (Table 3). Parameters listed in Table 2 but not in Table 3 could not be used due to skewed distribution or strong correlation with another parameter.

**Table 3:** Overview on the (G)LMs and their sub-models built for answering Questions Q1-Q5 and the corresponding training sections online dog training, practical seminar sessions and field test. Parameters listed in Table 2 but not mentioned here had to be excluded due to skewed distribution or correlations. Sub-models, necessary to avoid overfitting, were built based on hypotheses. Parameter definitions can be found in Table 2. N refers to sample size, * symbolises interactions.

Question	Training section	Response	Hypothesis	Sub-model	Link function
Q1	Online dog training	De.ave.duration (N = 179)	Team	Team	Gaussian
			Basic	Chosen alert behaviour + Cohort + Dog age + FCI + Team experience + Reward type	
			Training	Distraction + Sample odour + Target species + Training step	
Q2	Online dog training	Goal achievement (N = 203)	Team	Team	binomial
			Basic	Chosen alert behaviour + Cohort + Dog age + FCI + Team experience + Reward type	
			Training	De.ave.duration + Distraction + Sample odour + Training step + Target species	
Q3	Practical seminar sessions	Detection (N = 93)	On-site conditions	Cohort + FCI + Sample condition*Sample length + Target species + Test session +Wind	binomial
			Training conditions	Dried + Fresh_training + Plant parts + Participation online training seminar live + log(Search area) + Single blind + Steps skipped + Team experience + Training days/week	
Q4	Practical seminar sessions	Team score (N = 23)	On-site conditions	Cohort + Diff_goal + Percentage of fresh samples + Rel_goal + Wind	Gaussian
			Training conditions I	Dried + Fresh_training + Plant parts + log(Search area) + Reward type	
			Training conditions II	Participation online training seminar live + Session duration + Steps skipped + Team experience + Training days/week	
Q5	Field test	Field test conducted (N = 23)	Team performance	Diff_goal*Cohort + Rel_goal + Team score	binomial
			Motivation	Career + Community + Conservation + Science + Self-efficacy + Qualification	

For the test sessions of the practical seminar, we determined (Q3) which parameters influenced the detection of the plant samples, using a binomial error distribution (1 = sample found, 0 = sample not found). Only plant samples passed by the dog were included in the response variable. We further determined (Q4) the overall performance of the teams using the team score as response variable and a Gaussian error distribution. As conditions during test sessions were comparable between cohorts, we analysed all teams together. Explanatory parameters included social parameters, on-site conditions and training parameters as well as performance values from the online training averaged per team (Table 2). Again, two sub-models were created for Q3 and three sub-models for Q4 (Table 3) to avoid overfitting, and parameters listed in Table 2 but not in Table 3 could not be used due to skewed distribution or strong correlation with another parameter. Regrettably, it was not possible to add the team as a random intercept to Q3 models due to the low number of observations per team (3–12) in relation to the high number of teams (24).

For the field test, we determined (Q5) whether training pace and training goal achievement during online training and overall performance (team scores) during the practical seminar influenced whether a team conducted the field test using a binomial error distribution (1 = field test conducted, 0 = field test not conducted). We additionally investigated whether a team’s motivation for contributing to this project influenced whether the team conducted the field test. Thus explanatory parameters included performance values from online training and practical test sessions as well as motivation scores (Table 2). Two sub-models were created to avoid overfitting (Table 3). Only 23 teams provided all necessary information for this analysis.

All analyses were conducted in R version 4.3.3 [40]. All models were analysed and evaluated according to the following procedure. To avoid collinearity, we conducted a correlation test among all parameters in the respective (sub-)models and chose a conservative correlation threshold of R ≥ 0.4 upon significance. Further, the general variance inflation (GVIF1/2df) factor was calculated with all remaining parameters to ensure that no further collinearity was present in the data, which was determined as GVIF1/2df < 5 [41]. We then compared different initial parameter characteristics (such as interactions or polynoms) per sub-model using Akaike’s Information Criterion (AIC) [42] to find the full models, whereby AIC > 2 indicated statistically different models. Further, all possible model combinations were fitted within the given set of test predictors from the full models [43] using the R-package MuMIn [44] to identify the best model. Parameter estimates and standard errors were derived from the full models [45]. The relative importance of the parameters (sum of weights (sw)) was calculated by summing their AIC weights across all models. P-values were derived from likelihood-ratio tests (LRTs) comparing the full model to a model without the specific parameter, using F-tests for gaussian and Chi-Square-Tests for binomial error distributions. Models were validated through visual inspection of residuals for Gaussian error distribution and through testing dispersion, overdispersion, convergence and residuals for binomial error distribution. No deviations from model assumptions were detected. All model visualisations were created using the R-packages ggeffects [46], ggplot2 [47] and ggpubr [48]. Further R-packages used during data preparation, analyses and validation are referred to in the R-code, which is available together with all data through https://doi.org/10.5281/zenodo.21373572.

## Results

### Overall completion of the training programme

While we started with 30 teams initially, results are shown for 31 human-dog-teams because Team 70034 in cohort 1 continued in cohort 2 with their second dog as additional participant (Team 79430), training the dog independently and without documenting online training. Due to empty spots in the practical seminar of cohort 2, as other people had dropped out, they could participate and also conducted the field test. Team 79313 also completed the practical seminar, but had to cancel afterwards due to the dog falling ill. This team independently trained a second dog (Bavarian Mountain Hound) on *Ambrosia psilostachya*, which appeared to be more prevalent in their region than *A. artemisiifolia*. Although the team completed the field test with the second dog, we do not count them separately because training was not documented for the second dog and could thus not be analysed in detail. We only included this team in the analysis of whether motivation influences whether a team performed a field test (Q5, Motivation hypothesis), as the handler conducted the field test (albeit with the second dog).

Of 31 teams in total (Table 4), 27 (87.1%) completed online training, 13 from cohort 1 and 14 from cohort 2. Of them, 24 completed the practical seminar (77.4% of the total). Finally, 12 teams (38.7% of the total) completed the field test (including one non-passed). In total, 5 teams were trained on *A. artemisiifolia*, 11 on *I. glandulifera*, 12 on *F.* spp. complex*.*, and three did not reach the target scent imprinting step. Results shown here represent the status during testing and not the final performance of individual teams. Teams that left the project stated that they did so for personal reasons and not because of the training or project.

**Table 4:** Overview on volunteer detection dog teams, their dog breed and performances in the different training sections, associated to the two cohorts. NA symbolises that the team did not finish this training part. Diff_goal – Mean of the deviation from average duration to reach a training goal, in days (i.e., the training pace of a team; negative: faster than average, positive: slower than average); Rel_goal – Relative goal achievement in relation to the number of training steps filmed per team. *F. spp.* includes *F. japonica*, *F. sachalinensis* and their hybrid, *F.* × *bohemica*; IG = *I. glandulifera*; AA = *A. artemisiifolia*. P = passed; NP = Not passed.

Team ID	Cohort	Dog breed	Target species	Online training		Practical seminar		Field test
				Diff_goal	Rel_goal	Proportion detected	Team score	
69980	1	Longhaired Weimaraner	*F. spp.*	−16.4	88.89%	NA	NA	NA
70021	1	Malinois	*IG*	2.4	50.00%	0.5	3.5	P
70033	1	Magyar Viszla	*F. spp.*	4.7	100.00%	0.5	4.5	P
70034	1	Lapinporo- koira	*IG*	−25.7	90.00%	1	9	P
70035	1	German Shepherd	*IG*	13.0	33.33%	NA	NA	NA
70037	1	Mix	*F. spp.*	43.5	90.00%	0.6	5.4	P
70038	1	Mix	*IG*	−12.8	100.00%	0.75	6.75	NA
70039	1	Welsh Corgi Cardigan	*AA*	11.5	71.43%	1	8	NA
70040	1	Mix	*IG*	−6.9	81.82%	1	8	NA
70044	1	Lagotto Romagnolo	*AA*	6.5	100.00%	0.75	6.75	P
70048	1	Swiss White Shepherd – Husky Cross	NA	NA	NA	NA	NA	NA
70063	1	Nova Scotia – Golden Retriever Cross	*F. spp.*	7.0	100.00%	0.67	5.33	NA
70068	1	Mix	*F. spp.*	−29.8	100.00%	0.75	6	P
70069	1	Magyar Viszla	*F. spp.*	44.6	66.67%	0.8	7.2	NA
70074	1	Dalmatian	NA	NA	NA	NA	NA	NA
78724	2	Miniature Schnauzer	*F. spp.*	25.3	87.50%	0.33	1.67	P
78759	2	Labradoodle	*AA*	−25.1	100.00%	0	0	NA
78924	2	Lagotto Romagnolo	*F. spp.*	43.3	100.00%	0.33	1.67	NP
79115	2	Jack-Russel-Mix	NA	NA	NA	NA	NA	NA
79118	2	German Shepherd -Mix	*IG*	−36.9	100.00%	0.5	2.5	NA
79309	2	Pointer -Mix	*IG*	−54.5	100.00%	0.6	5.4	P
79313	2	Wire-haired dachshund	*AA*	−31.5	100.00%	0.67	3.33	(P*)
79316	2	Tervueren	*IG*	0.1	87.50%	0.6	5.4	NA
79322	2	Long-haired collie	*IG*	57.5	75.00%	0.25	2	P
79341	2	Lagotto Romagnolo	*AA*	35.1	100.00%	0.25	2	NA
79362	2	Czechoslovakian Wolfhound	*IG*	−7.7	50.00%	0	0	NA
79372	2	Cairn Terrier	*F. spp.*	113	66.67%	NA	NA	NA
79374	2	Mix	*F. spp.*	−70.4	100.00%	0.25	1.25	NA
79422	2	Golden Retriever – Catahoula Cross	*F. spp.*	22.4	83.33%	0.33	1.67	NA
78716	2	Weimaraner	*IG*	NA	NA	NA	NA	NA
79430	2	Laika-Mix	*F. spp.*	NA^+^	NA^+^	0.5	4	P

*this dog had to retire due to health issues, but passed with second dog trained identically. ^+^trained as second dog, all sections completed, but online training not properly documented

### Online dog training assessment

In cohort 1, 13 teams completed online training consisting of 12 main training steps, leading to a total of 156 training steps. However, 47 of these training steps were not sufficiently documented, leaving 109 training steps where we conducted video analyses. In cohort 2, again 13 teams completed online training consisting of 8 main training steps due to adjustments made by the trainers, leading to a total of 104 training steps. Of those, 10 training steps were not sufficiently documented, leaving 94 training steps where we conducted video analyses.

On average, it took 21.6 days (SD = 12.1) and 29.3 days (SD = 25.8) to reach the next training step for cohort 1 and 2, respectively. Despite the different training plans and some insufficiently documented training steps, overall duration was not significantly different between cohorts (paired t-test, p = 0.16). However, the timeline created for all teams and each training step, which graphically depicts the period of target achievement to scale in relation to the average date, shows that deviations around the average date were much larger for cohort 2 than for cohort 1 (Fig 2).

**Fig 2:**
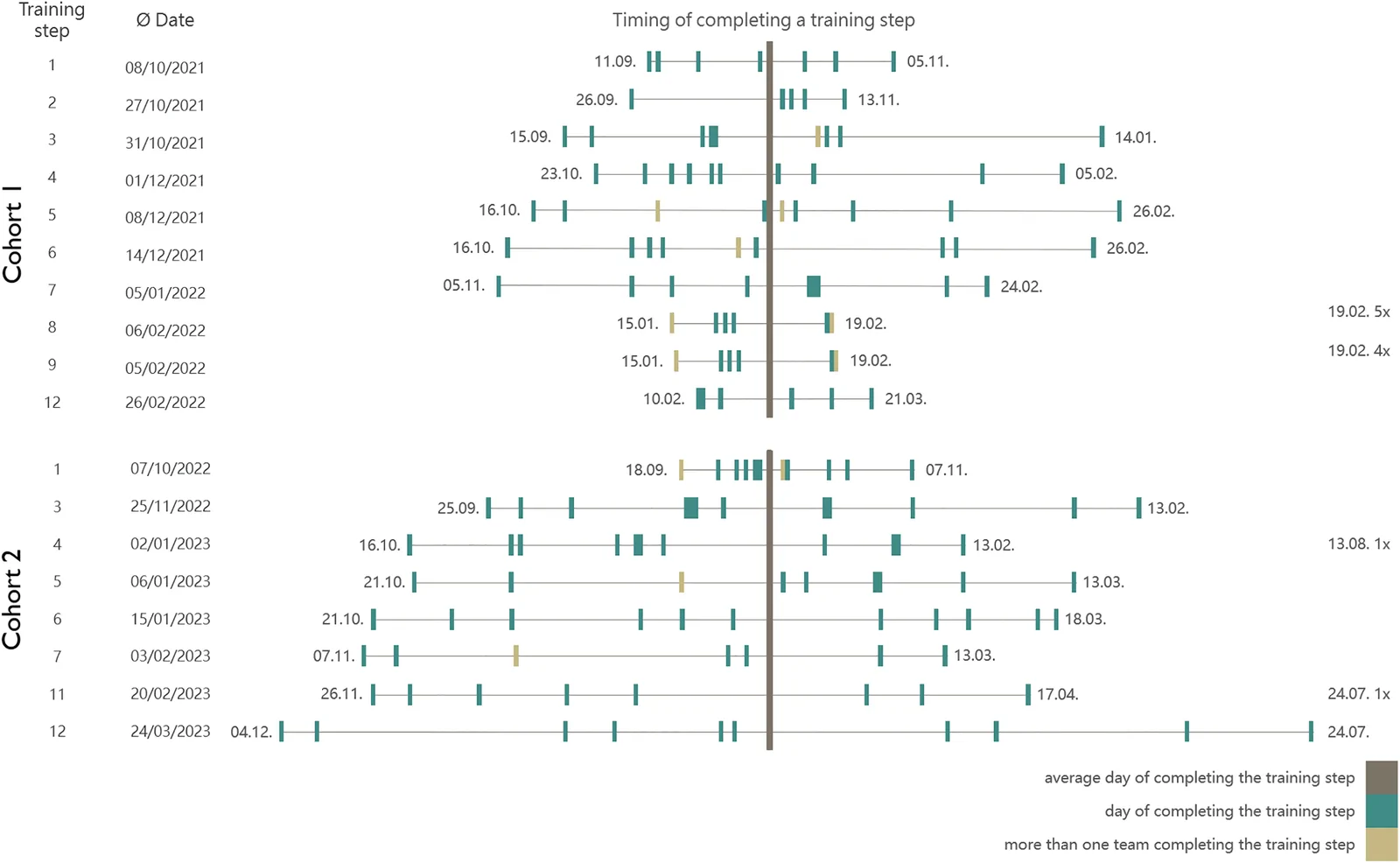
Timeline of training progress. Timing of completing training steps for all teams in cohort 1 and cohort 2 during the online training (Phases 1 and 2, excl. practical seminar tests). The figure displays the deviation (in days) from the average date each training step was completed (goal achieved = yes) across all teams. Minimum and maximum completion dates per training step are indicated. Project start dates were 11/09/2021 for cohort 1 and 17/09/2022 for cohort 2. Training steps correspond to those listed in Table 1.

Of the 109 and 94 training steps video-analysed for cohort 1 and 2, respectively, the training goal was achieved 94 (86.24%) and 85 (90.4%) times. In all cases except one, the goal was not achieved because the minimum alert duration was not reached, meaning that the alert was rewarded too early or interrupted by the dog. No false positive alert was observed. Both the training pace as deviation from average date to reach a training goal (Q1) and whether a training goal was achieved (Q2) were highly variable among teams (Hypothesis ‘Team’, Table 5). Model results of the Hypothesis ‘Basics’ (Table 5) showed that while cohort 2 was slower in reaching the training steps for the passive alert training (sw = 0.95), no differences occurred for achieving the training goals (sw = 0.33) (Fig 3). Instead, the FCI group of the dog was associated with both outcomes with dogs from FCI groups 6 (Scent hounds) and 7 (Pointing Dogs) reaching goals earlier than average and dogs from group 3 (Terriers) later than average (sw = 1). Less differences occurred for reaching the training goal, where dogs from groups 1 (Sheepdogs and Cattledogs) and 3 did achieve the training goal less often and dogs from groups 6 and 8 (Retrievers, Flushing Dogs, Water Dogs) always achieved the goal (sw = 1). Teams that chose ‘lay-and-stare’ as alert behaviour were associated with later goal achievement than those that chose ‘sit-and-stare’ or ‘freeze-and-stare’ (sw = 1, Fig 3A), and teams that chose ‘lay-and-stare’ slightly less often achieved the training goal (sw = 0.78, Fig 3F). Teams that used both toy and food as rewards were associated with earliest goal achievement, followed by those that chose toy only and food only as reward (sw = 0.99, Fig 3B). Moreover, selecting both toy and food as rewards was associated with a higher probability of goal achievement than selecting either reward alone (sw = 0.82, Fig 3G). Also, previous team experience was associated with training pace (sw = 0.99, Fig 3C) but not with goal achievement (sw = 0.43, Fig 3H), with those with experience in scent matching or object search being associated with earliest and those with experience in tracking with latest goal achievement. There was a trend that teams with experience in tracking more often missed the goal of a training step than the others, but confidence intervals overlap strongly. Likewise, dog age was associated with a higher-than-average training pace (sw = 0.63), which did not translate in accuracy of goal achievement (sw = 0.40).

**Table 5:** Parameter estimates and their standard errors (SE) obtained from the full models, importance values based on whole-model comparisons, and p-values obtained from LRTs of the full model against the model without the parameter in question. Estimates and standard errors of the multi-factorial predictors Team and FCI refer to the first factor which is displayed in brackets; those of other multi-factorial predictors are listed separately. Models refer to the question and the hypothesis according to Table 3. Parameter definitions can be found in Table 2. Importance values above 0.5 and p-values up to 0.09 are highlighted in italics. “:” symbolises interaction.

Model	Predictor	Estimate	SE	Importance (sw)	p-value
**Q1 - Team**	Team (70021)	0.70	0.41	*1*	*<< 0.001*
**Q1 - Basics**	Chosen alert behaviour (sit)	−2.95	0.38	*1*	*<< 0.001*
	Chosen alert behaviour (freeze)	−2.69	0.35		
	Cohort	0.50	0.17	*0.95*	*0.004*
	Dog age	0.06	0.03	*0.63*	*0.09*
	FCI (group 1)	0.86	0.23	*1*	*<< 0.001*
	Team experience (0)	−0.31	0.31	*0.99*	*0.0003*
	Team experience (2)	0.21	0.20		
	Team experience (3)	−0.99	0.24		
	Reward type (toy)	−0.56	0.31	*0.99*	*0.001*
	Reward type (both)	−1.29	0.34		
**Q1 - Training**	Distraction	0.30	0.13	*0.82*	*0.02*
	Sample odour	0.05	0.15	0.28	0.75
	Target species (*F. spp.*)	0.09	0.20	*0.98*	*0.003*
	Target species (*I. glan.*)	−0.48	0.20		
	Training step	−0.03	0.03	0.39	0.25
**Q2 - Team**	Team (70021)	−2.01	1.62	0.30	*0.003*
**Q2 - Basics**	Chosen alert behaviour (sit)	20.18	2625.96	*0.78*	*0.009*
	Chosen alert behaviour (freeze)	2.33	1.31		
	Cohort	0.10	0.95	0.33	0.92
	Dog age	0.13	0.14	0.40	0.33
	FCI (group 1)	−1.99	0.92	*1*	*0.0002*
	Team experience (0)	−0.51	1.49	0.43	0.16
	Team experience (2)	−1.14	0.95		
	Team experience (3)	1.00	1.05		
	Reward type (toy)	−0.03	1.11	*0.82*	*0.098*
	Reward type (both)	2.84	1.50		
**Q2 - Training**	De.ave.duration (scaled)	−0.57	0.25	*0.77*	*0.02*
	Distraction	−1.03	0.36	*0.97*	*0.003*
	Sample odour	0.32	0.50	0.33	0.52
	Target species (*F. spp.*)	0.20	0.89	*0.69*	*0.04*
	Target species (*I. glan.*)	−1.20	0.82		
	Training step	0.11	0.09	*0.50*	0.22
**Q3 - On-site conditions**	Cohort	−2.12	0.84	*0.97*	*0.006*
	FCI (group 1)	−0.36	0.93	0.03	0.891
	Sample condition	5.68	3.87	*0.65*	*
	Sample length	0.86	0.40	*1.00*	*
	Sample condition: sample length	−0.57	0.40	0.48	*0.068*
	Target species (*F. spp.*)	−2.08	1.55	*0.71*	0.315
	Target species (*I. glan.*)	−0.39	1.16		
	Test number (2)	0.30	0.97	0.13	0.924
	Test number (3)	0.07	1.13		
	Wind (slight)	−0.14	1.43	0.32	0.806
	Wind (intermittend)	0.33	1.46		
**Q3 - Training conditions**	Dried	0.83	0.81	0.30	0.289
	Fresh_training	−1.00	0.78	0.36	0.191
	Plant_parts (3)	1.72	1.01	*0.77*	*0.009*
	Plant_parts (4)	3.07	1.11		
	Participation_online training seminar_live (partly)	0.54	1.03	0.25	0.448
	Participation_online training seminar_live (yes)	1.37	1.14		
	log(Search_area)	−0.03	0.22	0.29	0.897
	Steps_skipped (many)	−0.82	0.96	*0.59*	*0.057*
	Steps_skipped (none)	−2.73	1.30		
	Team experience	0.11	0.35	0.36	0.763
	Training days/week	0.18	0.44	0.29	0.682
	Single_blind (none)	1.70	1.08	0.13	0.342
	Single_blind (many)	0.61	0.80		
	Single_blind (most)	1.67	1.07		
**Q4 - On-site conditions**	Cohort	−4.12	0.71	*1.00*	*<0.001*
	Diff_goal	0.01	0.01	0.28	0.504
	Percentage of fresh samples	−1.90	1.50	0.43	0.139
	Rel_goal	0.02	0.02	0.33	0.307
	Wind (slight)	−4.38	1.79	*0.90*	*0.013*
	Wind (intermittent)	−3.39	1.86		
**Q4 - Training conditions 1**	Dried	−0.65	1.29	0.31	0.676
	Fresh_training	−0.56	1.20	0.28	0.811
	Plant parts (3)	2.55	1.48	*0.76*	0.165
	Plant parts (4)	3.21	1.58		
	log(Search_area)	0.28	0.31	0.29	0.721
	Reward type (food)	0.63	1.22	0.13	0.843
	Reward type (toy)	−1.65	1.22		
**Q4 - Training conditions 2**	Participation_online training seminar live (partly)	−0.29	2.20	0.14	0.990
	Participation_online training seminar live (yes)	−0.15	2.01		
	Session_duration	0.14	0.07	*0.78*	*0.062*
	Steps_skipped (few)	3.26	1.84	*0.71*	0.221
	Steps_skipped (many)	2.90	2.14		
	Team experience	0.49	0.65	0.46	0.459
	Training days/week	0.30	0.67	0.31	0.666
**Q5 - Team performance**	Cohort	−1.39	1.64	0.39	*
	Diff_goal	−0.01	0.03	0.39	*
	Diff.goal: Cohort	0.04	0.03	0.07	0.27
	Rel_goal	0.02	0.03	0.30	0.55
	Team score	−0.11	0.30	0.29	0.72
**Q5 - Motivation**	Career	0.57	0.60	0.39	0.28
	Community	2.14	1.59	*0.58*	*0.04*
	Conservation	−2.10	1.35	*0.54*	*0.04*
	Science	2.92	1.45	*0.89*	*0.003*
	Self-efficacy	−1.09	0.77	0.40	*0.09*
	Qualification	−0.36	0.64	0.33	0.56

* Missing p-values are not indicated because they are conditional on another predictor and thus do not have a meaningful interpretation.

**Fig 3:**
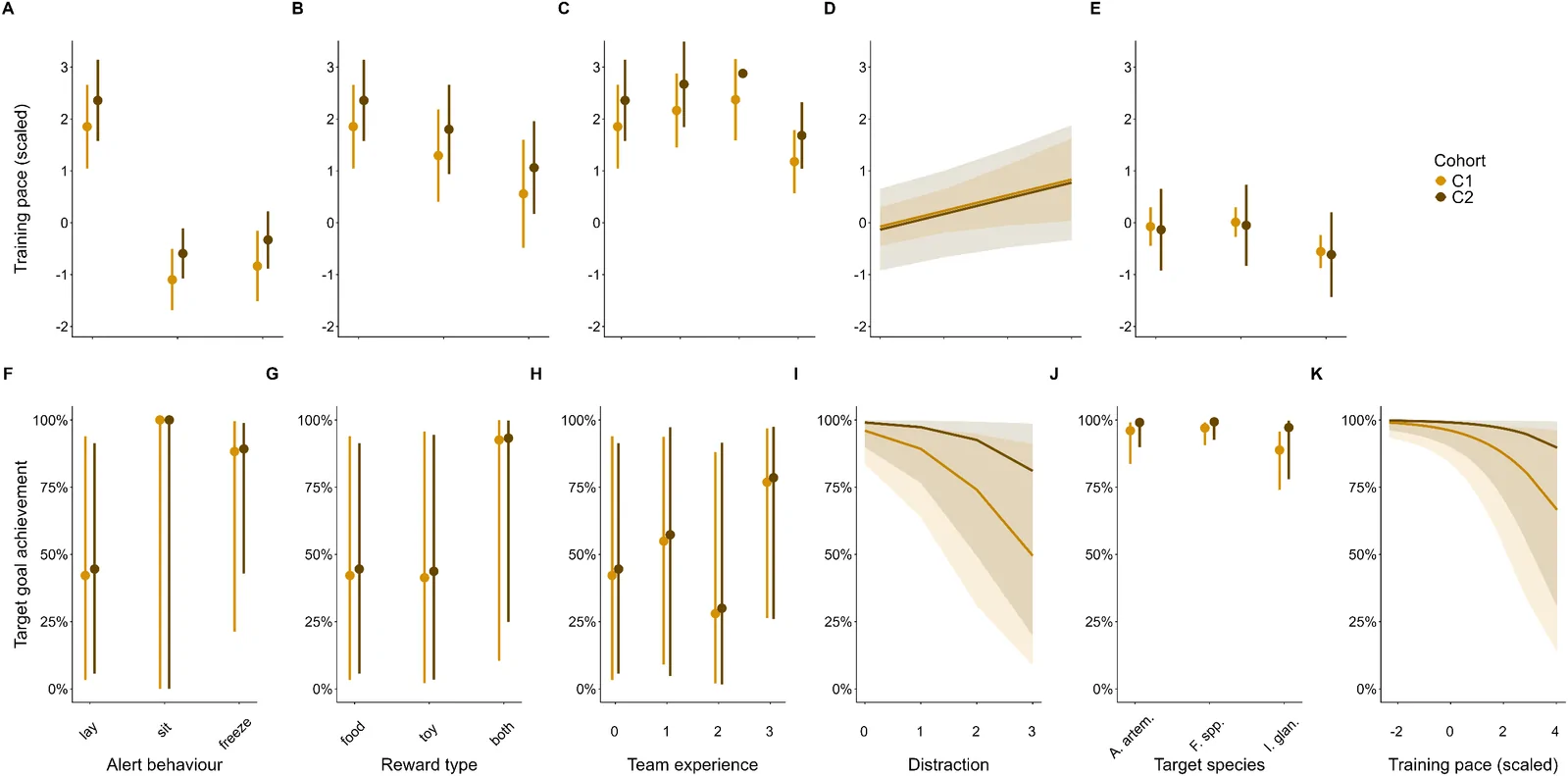
Parameters associated with training pace and target goal achievement per cohort during online training. Training pace (upper row) as difference from the average date when the target goal of a training step was achieved (positive: slower than average, negative: faster than average), scaled per cohort to a mean of 0 and a standard deviation of 1, as well as likelihood of target goal achievement in percent (lower row) in relation to the most important model parameters. A/F – Alert behaviour; B/G Reward type; C/H Team experience (0 = none, 1 = dog sports, 2 = tracking and general scent work, 3 = scent matching or object search); D/I – Distraction; E/J – Target species (A. artem = *A. artemisiifolia*, F. spp. = *Fallopia spp. complex*, I. glan = *I. glandulifera*); K – Average training pace. Graphs are separated per cohort. Parameter definitions can be found in Table 2.

Model results of the hypothesis ‘Training’ (Table 5) revealed that more distractions were associated with higher-than-average training pace (sw = 0.82, Fig 3D) and a decreasing likelihood of achieving the target goal (sw = 0.97, Fig 3I). Moreover, the target species influenced training pace and target goal achievement. Specifically, *I. glandulifera* yielded in lower-than-average training pace (sw = 0.98, Fig 3E) but also a slightly decreased probability of goal achievement (sw = 0.69, Fig 3J). However, the manipulation of the sample odour did not affect training pace or goal achievement, indicating no causal effects of sample odour (hard rubber toy (aka KONG) or a plant) on performance of the passive alert training. No other parameter influenced training pace, but a trend occurred that with training, teams became more accurate with an increase in the likelihood of achieving the training goal (sw = 0.50). Also, teams that reached training steps faster-than-average were associated with a higher likelihood to achieve the training goals (sw = 0.77, Fig 3K). An overview on best models according to model selection can be found in S5 Table.

### Practical seminar

A total of 72 trials (3 test sessions x 24 teams) were planned, each with one or two plant samples laid out. However, as the teams could decide to skip a search or terminate it before completion, only 93 samples out of 107 planned could be included in the analysis. Team-specific proportions of detected plants (Q3) ranged from 0 to 100%, with an average of 53.9% (standard deviation (SD) = 28.3) in total, 75.6% (SD = 18.5) for cohort 1 and 35.5% (SD = 21.3) for cohort 2 (Table 3). Of the dogs that had not previously used fresh samples in training (N = 9), 5 were able to immediately detect a fresh plant laid out in the practical seminar, resulting in a spontaneous generalisation rate of 56%. No false positive alerts were observed. As Team 79430 could not document online training, analyses based on hypothesis ‘Training’ were conducted for the remaining 23 teams.

Regarding the hypothesis ‘On-site conditions’ (Q3, Table 5), sample length had the strongest effect on detection probability across all teams (sw = 1, Fig 4A), with larger plant parts resulting in higher detection probabilities (Table 5). Detection probability was higher in cohort 1 than in cohort 2 (sw = 0.97, Fig 4A). Furthermore, sample condition (sw = 0.65) affected detection probability, with thawed samples resulting in higher detection probabilities than fresh ones. This effect was particularly strong in interaction with sample length (sw = 0.48, Fig 4A). Target species (sw = 0.71) also affected detection probability, with dogs trained on *A. artemisiifolia* showing higher detection probabilities than those trained on *I. glandulifera* and *F.* spp. complex.

**Fig 4:**
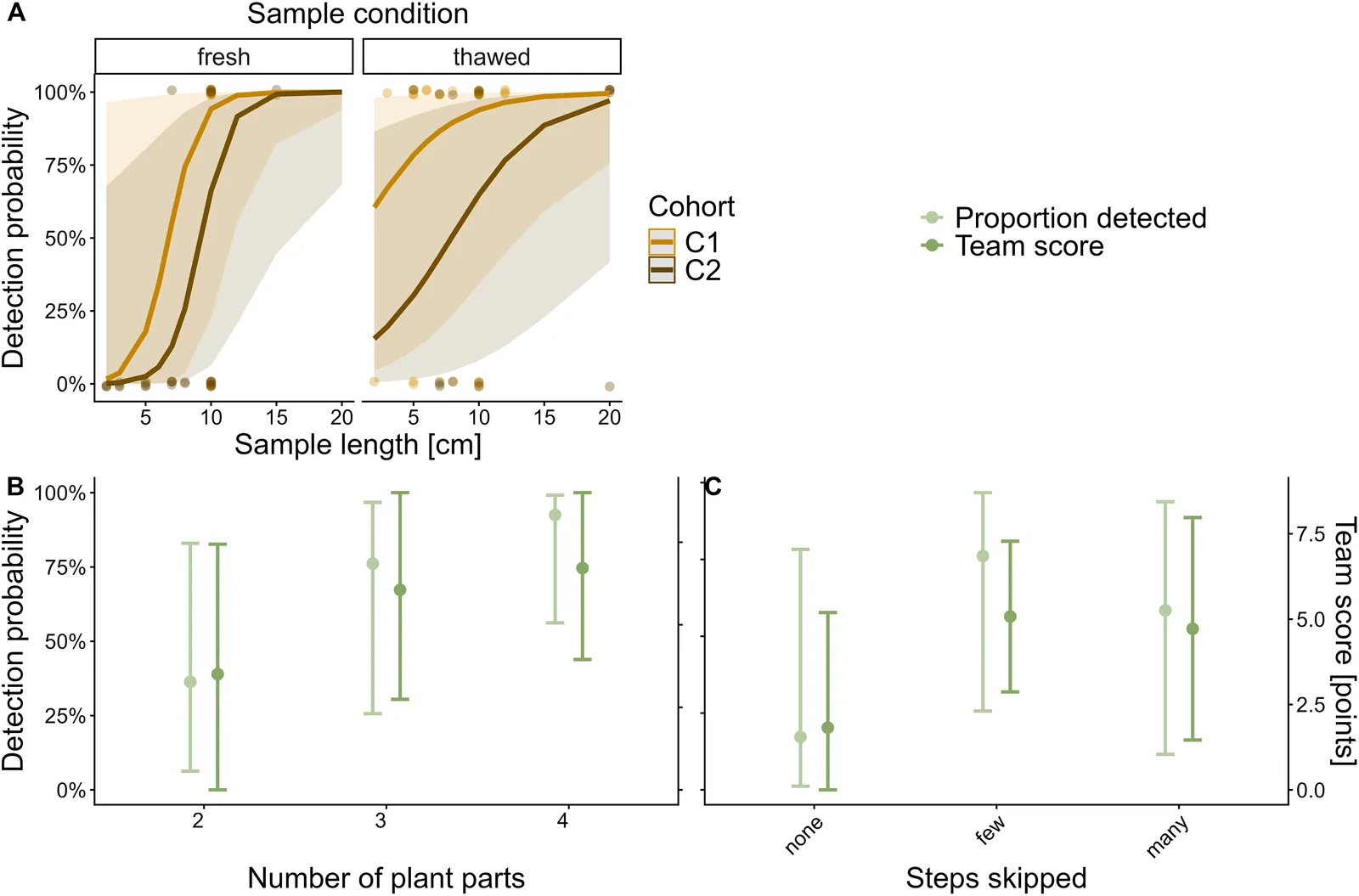
Parameters influencing success of the practical seminar tests. Predicted detection probabilities of plant samples (A-C), team scores (C) and their 95% confidence intervals (shaded areas/bars) across all teams in relation to the most important ‘on-site’ (A) and ‘training (B,C) parameters’ during the practical seminar tests, separated per cohort for A.

Parameters from the hypothesis ‘Training conditions’ associated with detection probabilities (Q3, Table 5) included the number of plant parts used for training (sw = 0.77, Fig 4B) and number of steps skipped (sw = 0.59, Fig 4C). Highest detection probabilities were observed among participants who included all plant parts (leaves, stem, roots and flower) in their training (Fig 4B) and skipped training steps based on their level of proficiency (Fig 4C).

The overall performance of the teams according to team scores (Q4) averaged 4.2 points (SD = 2.6), with cohort 1 and 2 averaging at 6.4 (SD = 1.65) and 2.4 (SD = 1.7) points, respectively. According to the hypothesis ‘On-site conditions’ (Table 5), team scores were higher in cohort 1 than cohort 2 (sw = 1.00). Team scores were also associated with wind, whereby highest team scores were observed under no wind (sw = 0.90). A higher proportion of fresh plant samples only slightly reduced team scores (sw = 0.43). Performance of teams during online training did not affect performance during test sessions (Table 5).

As explained above, models regarding training conditions from previous online training were split into two sub-model due to the small sample size (N = 23) (Table 3). Regarding hypothesis ‘Training conditions 1’ (Q4, Table 5), highest team scores were observed among participants who included all plant parts (leaves, stem, roots and flower) in their training (sw = 0.76, Fig 4B). Analyses regarding hypothesis ‘Training conditions 2’ (Q4) revealed that higher team scores were associated both longer training session durations (sw = 0.78) and skipping steps (sw = 0.71) in the training plan (Fig 4C). An overview on best models according to model selection can be found in S5 Table.

### Field test

Of the 12 teams that conducted the field test, 11 teams passed (Table 4) with an average proportion detected of 74.7% (excluding one team that did not pass). In the first cohort, 6 teams (40%) conducted the field test with an average proportion detected of 71.7% [50–100%]. In the second cohort, another 6 teams (37.5%) conducted it with an average proportion detected of 63.4% [0–100%], respectively, excluding one team that did not pass, 79.3% [57.1–100%]. Again, no false positive alerts were observed. As the teams carried out the field test independently, the samples varied considerably in length and number, so that the proportion detected was not analysed further. We rather focused on analysing whether a team conducted the field test at all. Higher proportions of teams with higher previous experience were observed to complete the field test, whereas teams without any prior experience did not complete the programme. However, this pattern was not observed when cohort 2 was examined alone. Teams 79430 and 79313 (both cohort 2) were omitted from hypothesis ‘Team performance’ as the first person was part of the first cohort with a different dog (that also passed the field test) and online training data was not available for the second dog, and the second person conducted the field test with a different dog than the online training and practical seminar due to this dog falling ill (Table 4).

Neither the team performance during online training nor during the practical seminar were associated with whether a team took part in the field test or not (Q5, Hypothesis ‘Team performance’, Table 5). However, the hypothesis ‘Motivation’ revealed that a highrer interest in both science (sw = 0.89, Fig 5A) and community (sw = 0.58, Fig 5B) were associated with a higher prrobability of a team conducting the field test. However, a higher motivation in conservation was associated with a lower probability of a team conducting the field test (sw = 0.54, Fig 5C). Another trend showed that an increased motivation in self-efficacy was associated with a lower probability of conducting the field test (sw = 0.40, Fig 5D, Table 5). An overview on best models according to model selection can be found in S5 Table.

**Fig 5:**
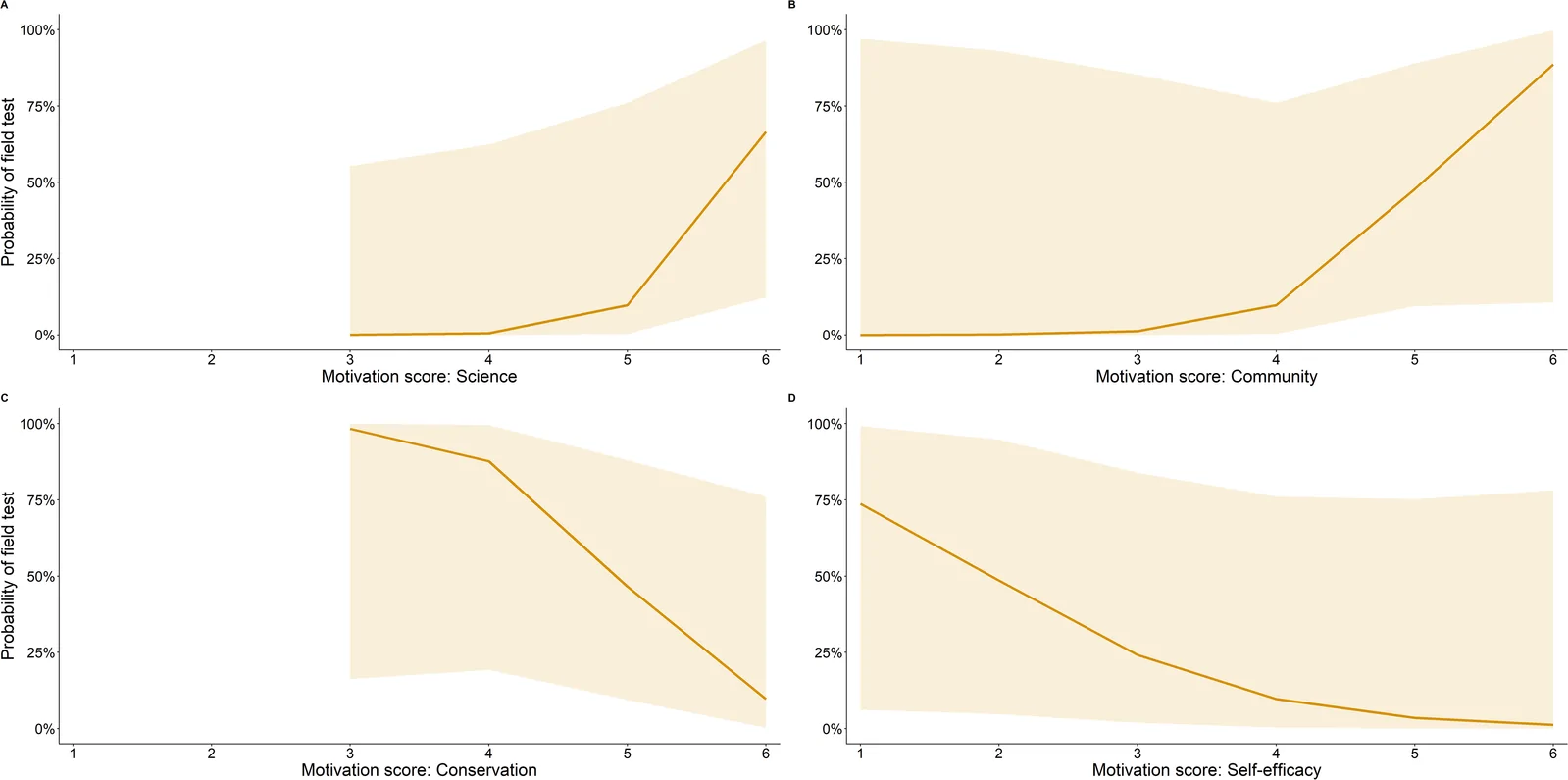
Association of the motivation score in different disciplines on the probability to conduct the final field test. Shaded areas refer to the 95% confidence intervals, teams of both cohorts were included. Scores between 1 and 6 are possible, whereby higher scores refer to higher motivation in that discipline.

## Discussion

The results show that it is generally possible to train volunteer WDD teams to detect invasive alien plants, enabling dogs to identify target species reliably. Participation in such projects usually provides dogs with considerable mental stimulation and enrichment, thereby strengthening the relationship between dog and owner [49]. In addition, due to the popularity of dogs, WDD teams offer excellent opportunities for public outreach and engagement [50], especially for underrepresented topics such as invasive alien plant species. Despite the benefits, successful outcomes required intensive training that was difficult to incorporate into daily routines. As hypothesized – and consistent with the only two comparable large-scale volunteer WDD studies to our knowledge [19, 33, 38, 51, 52] – engagement and performance varied considerably among teams, highlighting variation in motivation and capacity among participants. Participants who completed the training also reported that the training was more time-consuming than they had originally thought. Importantly, teams that fall substantially behind schedule are unlikely to catch up, leading to a persistent increase in training pace over time. In this study we aimed at investigating factors affecting performance of the volunteer WDD-teams during different training sections.

The first training phase was conducted as online training, with the overall goal of teaching the teams passive alert behaviour and then simple search procedures for their target scent. Performance during online training was primarily affected by inherent social factors and reward types used. In contrast, whether starting with a substitute odour (hard rubber toy) or the target plant had no measurable effect on outcomes. Our adjustment to start cohort 2 with the target odour instead of a substitute odour was a response to feedback from cohort 1. It was intended to accelerate the transition to actual odour search training (plants) and allow for a longer period of supervised training with the target odour, but it had no impact on team performance.

Instead, dogs performing a lay-and-stare alert behaviour (N = 16, 2 dogs) were observed to be slower and less accurate in comparison to sit-and-stare (N = 16, 2 dogs) or freeze-and-stare alert behaviours (N = 171, 22 dogs). As teams usually did not reach the training goal because the alert duration (staring) was not long enough according to the training steps in the training plan, we suspect that forming the alert behaviour in a laying position is more complex than in a sitting or standing position. Indeed [53], showed that the alert behaviour may influence detection performance, however, they did not test lay behaviour and the number of dogs was very low (4 dogs). Although the number of dogs and trials is higher in our sample, most handlers have chosen the freeze-and-stare alert behaviour and results of lay and sit alerts could thus also reflect training of the individual teams over a general pattern. Moreover, the alert behaviour did not affect the WDD-Team performance in subsequent outdoor searches, where the alert duration was never measured and the lay behaviour was a precise enough sign to the handler to find the plant eventually.

An equally important initial factor was the type of reward, with the use of toys (N = 22, 3 dogs) rather than food (N = 165, 21 dogs) being associated with an enhanced performance of training pace, but alternating the use of food and toy rewards (N = 16, 2 dogs) being associated with fasted goal achievement and highest accuracy. The use of toys was often seen as a high level of reward motivation increasing arousal and slowing the learning process [54, 55]. However, recent studies have shown that dogs with a higher arousal due to toy or play motivation (which must not be confused with over-arousal or a lack of arousal regulation) achieved higher scores in odour detection tasks [56, 57], and another study showed that using toys as reward instead of food reduced false alerts in highly-trained detection dogs [58]. In general, a play session following a training session can increase performance of dogs [59]. Although all participants tested for their dog’s preferred reward type initially [35], we assume that employing variable reward types – providing the dog with what it requires at a given moment and preventing that a reinforcer loses its effectiveness through satiation – will yield the highest training effectiveness. Again, confounding factors with individual training differences cannot be excluded and once the dog was properly trained, no effect of the reward type was found in the subsequent outdoor trials.

The effect of the teams’ previous experience varied slightly between the cohorts, but the teams with scent detection experience were fastest during online training and those with tracking experience were initially slower and less accurate, presumably because the training of tracking dogs differs strongly from the training of other sporting or scenting dogs, particularly in the search behaviour itself and cooperation with the dog handler. Moreover, teams with more experience tended to perform slightly (but non-significantly) better in the overall team score of the practical seminar tests, but differences were marginal. Likewise [51], showed that dogs with variable previous experience were able to learn detecting reptile scent, and both dog and handler experience also did not affect the training pace or proportions detected of indoor detection experiments [19]. However [60], found that dogs previously trained as search-and-rescue dogs performed slightly less accurate in detecting and discriminating target scents in comparison to dogs exclusively trained as WDD, suggesting that while it is undoubtedly possible, and, on an individual level, maybe even beneficial, to train a dog for different search tasks, it can become more complex and time-consuming to perform the different tasks accurately.

Although both cohorts exhibited comparable pace and accuracy during online training, cohort 1 performed better in the test sessions during the practical seminar, which then, however, did not translate into the proportion of teams conducting the final field test (6 per cohort). Although we can only speculate as to why this difference occurred, we had the impression that participants from cohort 2 showed increased tension during the tests, which may have impacted their on-site performance [61], ultimately affecting the dog’s performance [62], and possibly influencing other participants through emotional contagion [63]. Weather conditions that could have potentially been associated with performance were recorded inconsistently across test sessions by different observers and were not adequately standardized. Therefore, we refrain from drawing general conclusions, although wind – unlike other factors – may have had an effect.

A more tangible factor was that higher detection probabilities were achieved for larger plant parts, which likely emit more odour and are therefore easier to detect [6], and thawed than for fresh ones. Some dogs were confronted with fresh plants for the first time during these test runs, but another study found that dogs showed more frequent alerts for frozen or thawed targets compared to dried or live ones, regardless of prior training [15]. This might result from the processing method as some volatile organic compounds (VOCs) might decrease during the drying process, dependent on species [64], while freezing might change concentrations of VOCs, possibly leading to an increase of overall odour [65]. In our project, spontaneous generalisation rate from thawed to fresh plant parts for dogs that had never trained with fresh plants before was 56%, and thus much lower than reported previously [15]. Best results for detection and generalisation were observed when the participants included all parts of the plant (leaves, stem, roots and flower) in their training, which is consistent with the findings of [66], who found that generalisation was easier if dogs have been trained with a variety of samples or sample mixtures. Moreover, teams which were able to adapt the training plan according to their needs (i.e., skipping steps where necessary) performed better than those following the training plan strictly. The decisions as to whether a training step was skipped or not were occasionally made by experienced dog handlers themselves or initiated by the trainers. We assume that handlers skipped steps only after their dogs had mastered the preceding ones and that their familiarity with their dogs enabled them to accurately interpret behaviour and adapt the training plan accordingly (“Train the dog in front of you” [35],). However, progressing too quickly (skipping many steps) could, at some point, lead to poorer results [67], whereas progressing too slowly (not skipping any step) could also lead to frustration [35] or dogs losing interest [68]. It is also possible that teams that did not skip a step generally found it more difficult and therefore needed all the steps. For further studies, we recommend clearly defining the achievement of the training steps, but giving the freedom to adapt the training plans while standardising and monitoring the most important and broader training objectives. Finally, longer training duration was associate with improved team performance, which may reflect more focussed and concentrated training known to improve retention of learning in detection dogs [57]. Regrettably, we did not ask our participants whether they had or had not used marker training, where a signal that predicts a reward is used to provide immediate feedback for a correct response and to bridge the time between the desired behaviour and the reward. This might have affected both the training pace and accuracy during the initial training as well as the team performance in the practical tests on site [55].

Of the 12 teams conducting the field test, 11 teams passed with an average proportion detected of 74.7%, ranging from 50% (1 Team) to 100% (3 Teams). For logistical reasons, all but one field test was conducted by the participants (with helpers), and only the results were reported to us. This limitation must be considered when interpreting the proportions detected. Nevertheless, in a comparable study, outdoor detection rates of 69.78% were reported, and even teams that had indoor detection rates of as high as 94.12% dropped to 70% in simple and 60.61% in complex field conditions at first exposure. The variation in proportions detected among volunteer WDD-teams, both at the interim test (practical seminar) and the final test, was much higher than for professional WDD-teams [8,60,69,70], however, sample sizes in studies with professional WDD-teams are usually low (N = 2–4). Professional plant detection dogs achieved average proportions detected for different weeds of 88% in experimental settings and up to 100% in simple field conditions comparable to our tests [4], and three dogs trained on spotted knapweed (*Centaurea stoebe*) achieved proportions detected in outdoor searches of 85.7% [71] and 81.1% across different plant sizes [6]. However, professional WDD-teams underwent rigorous training and selection, as for example [8] excluded three out of five dogs before deployment and [72] developed a rigorous selection protocol for weed detection dogs. Although we initially included a selection procedure for the volunteer WDD-teams chosen, no further selection or exclusion procedure took place in order to reduce the tension of the participants and keep motivations high. However, dog and handler characteristics of professional WDD-teams may differ from that of non-working pet dogs, which could explain the large variability found in this study population [11, 56].

As individual field tests also varied due to the individual realisation, in this study we only investigated whether a field test was carried out. In contrast to our hypothesis, neither performance during online training nor during the practical seminar were ultimately associated with operational capability. Instead, personal motivation was the key factor in whether teams completed the full programme – rather than training progress or inherent social factors – as reflected by the fact that most field tests were conducted shortly after supervised training. Participants with higher motivation to support a scientific research project were observed to be more likely to conduct the field test, stating that they understood the importance of providing data for analyses. Also, higher motivation to support the community was associated with a higher probability to conduct the field test, while higher motivation in both conservation and self-efficacy were associated with a decreased probability. In comparison, in a citizen science project on frog biodiversity, both motivation in science and in conservation increased the willingness to contribute, but the motivation in science showed higher correlations [73]. However, the opposite was found in the Virginia Master Naturalist volunteer program [74]. An analysis of motivation in the UK Garden Wildlife Health program revealed that people conducting systematic monitoring had higher motivation in science than those conducting opportunistic surveys, but specifically conservation was not an important motivation in general. Instead, motivation for the community and values (in nature or science) were generally high, but also gaining knowledge was important, which we only indirectly asked our participants [75]. In contrast, in the agricultural project Soy in 1000 Gardens, retention in the project was not affected by self-efficacy (“Enhancement”) and community (“Social”), whereas participants that dropped off had a higher motivation in career than those that retained [76]. Our study observed a non-significant trend in the opposite direction, possibly because the category “Career” combined both professional WDD handlers and dog trainers, whose motivations may differ. A survey among participants of various citizen science projects tackling conservation issues revealed that community motivation led to higher engagement across projects, as well as gaining and sharing knowledge, but otherwise motivations differed among projects [77]. Importantly, caution should be taken when analysing and comparing motivation scores on Likert-scale because these data are ordinal, may not have equal intervals between response options, and are susceptible to response biases, limiting the appropriateness of certain statistical methods and cross-group comparisons [78].

Overall, while a total of 64.5% of our participants remained in the project until the end and 61.3% reported regular or occasional detections of wild IAS with their dogs (data not shown), only 38.7% actually completed the training. While the first rate of participants remaining in the project is comparable to the proportion found in another study training volunteer WDD-teams on-site [33], this same study also showed that only between 31.6% and 68.4% of the initial participants fulfilled all tasks including questionnaires. In comparison, 55.6% and 61.5% completed all experiments in [51], while another study on volunteers and IAS showed similar retention rates to our study (around 40% [79],). Moreover, retention rates are usually lower in online volunteer projects such as this one, although the alignment with hobbies (in this case, dog training) was thought to increase retention rates [74]. The same authors reported that typical reasons for participants to drop out were “time commitment; struggles with the online components; lack of tangible, real-world project impact; and limited social interactions” ([74], p. 6). Our participants primarily cited personal reasons for dropping out, such as relocations and changes in life circumstances.

Online training was the only viable approach to provide cross-regional training to volunteer WDD-teams at the scale required, providing the necessary flexibility in terms of timing and location to meet the needs of participants and the trainers, especially during the COVID-19 pandemic. However, almost all participants expressed a preference for more on-site training, suggesting that a more regional approach could be beneficial in future projects. In addition, the reliance on online training and session recordings required a high level of individual responsibility on participants to ensure data availability. The overall process was perceived as complex, and occasional technical issues with the online training platform may have contributed to frustration and reduced motivation, potentially resulting in incomplete data and some drop-outs.

Importantly, we set a rather low hurdle to pass the field test with a minimum proportion detected of 50% to decrease the tense and prevent exam anxiety. Furthermore, we provided those volunteer WDD-teams that passed the field test with a certificate to increase their motivation. However, another study found that neither gamification nor competition affected participation or retention of volunteers [73]. Instead, regular feedback may increase retention rates [80], which was positively acknowledged by the participants for phases 1 and 2. Although phase 3 included feedback, individual training, and an online discussion group, the intensity was lower than in phases 1 and 2. This may have also contributed to participants remaining in the project without completing the final field test. Another limitation was that, beyond theoretical input via webinars, the project did not sufficiently address the inhibition threshold related to independently conducting IAS monitoring and fieldwork—skills essential for later deployment. Closer supervision and more on-site support could have better prepared participants to recognise their target species, conduct basic fieldwork (e.g., transect walks), and interpret their dogs’ search behaviour. These abilities were later required to prepare the fieldwork and to evaluate the results of their dogs, potentially affecting the likelihood of conducting the field test as well. Further analyses on motivation of participants still need to be carried out. Moreover, future studies should also include questionnaires for the dog trainers about their general experience of participating in such studies, which might provide additional useful information on the feasibility of volunteer programmes like this one, and potentially options for improvements.

Finally, this pilot project seems to be one of the rare volunteer-based WDD projects with the precise aim to detect IAS in the field and not in experiments. The only other study where volunteer WDD-teams were used in real deployments, to our knowledge, was on an endangered stonefly species [52]. As hypothesised, we could confirm substantial variation in training success and performance among teams (hypothesis 1), and more effort regarding reward type, longer training sessions, more plant parts and adaptive training plans could improve performance (hypothesis 2). However, in contrast to hypothesis 3, successful completion of one phase of the training programme was not associated with a higher likelihood of successful performance in subsequent phases. Importantly, even after completing the training programme with promising results, maintenance is needed to keep high detection rates [81], although the dogs might generally remember their target odour quite some time after being without training or deployment [82]. Overall, it should never be underestimated that substantial time and financial costs are associated with training volunteers and they do not come for free [83], especially not when using a co-design approach [84]. Despite these costs, we expect that volunteer IAS detection dog teams have the potential to fill essential data gaps on IAS occurrence and spread. IAS do not only pose an environmental risk or affect human health, they are also an economic burden [85,86]. In Germany, for instance, these economic costs were conservatively estimated at US$ 9.8 billion between 1960 and 2020 [3]. The cumulative damage costs of IAS vary by species, but most of the taxa studied show no signs of cost saturation with increasing population density, but exponential growth in their associated costs [87]. Thus, the use of effective and economical detection methods is essential [1], particularly for EDRR management, and costs for a well-designed volunteer management including WDD seem to be well spent and are marginal compared to the expected damage costs.

## Recommendations for future projects

The following section outlines key recommendations for improving effectiveness and designing future projects involving volunteer WDD teams.

### Volunteer selection

#### Involvement of volunteers.

Completion of the training programme is likely associated with volunteers’ motivation in science and community, which can enhance during the programme, but will also depend on available time. We therefore recommend clearly communicating the expected time commitment, using a structured application process to assess motivation, and maintaining engagement by actively involving volunteers in data collection and the evaluation of results.

#### Previous experience.

Previous dog training experience may facilitate progression through the training programme, whereas experience in other search disciplines (e.g., tracking or trailing) may initially increase training demands. Recruiting teams with dogs that already have a trained alert behaviour could therefore reduce training effort. In addition, experience beyond detection dog work, such as ecological expertise, knowledge of the target species, or survey experience, is likely to benefit field deployments and data collection.

#### Dog selection.

Dogs selected for volunteer WDD work should be healthy, resilient, physically fit, and readily motivated by food and/or toys. As selection criteria may vary depending on the project, additional project-specific requirements should be considered and may differ from those used for professional WDDs [11].

### Initial training

#### Alert behaviour.

Alert behaviour should be tailored to the individual dog whenever possible. For WDD tasks, passive alerts are recommended to minimize disturbance to wildlife. Sit-and-stare and stand-and-stare alerts are generally suitable and can be selected according to the preferences of the dog, handler, or project requirements. Although lay-and-stare alerts may require more training initially, they appear to perform similarly in field deployments once reliably established, except where environmental conditions restrict the dog’s ability to perform the behaviour.

#### Reward type.

Dogs motivated by both food and toys may progress more easily through training, and incorporating play sessions may further support training success. These factors appear to primarily facilitate initial training and reduce time commitment rather than improve performance during field deployments.

#### Training plan.

A structured training plan with clearly defined mandatory milestones and flexible interim steps is essential to ensure consistent training while allowing adaptation to individual needs. Completion of mandatory (main) steps should be documented to monitor training progress. Video tutorials may further support participants in implementing key training stages.

#### Sample odour.

Starting passive alert training with either a substitute or the target odour appears not to affect subsequent dog performance; the choice should therefore be based on practical considerations such as target availability, legal restrictions, biosecurity, and the nature of the target. Once target odour training begins, exposure to a wide variety of samples (e.g., different sizes, conditions, locations, individuals, age classes, sexes, or plant parts) is recommended to promote generalisation and improve performance during field deployments.

### Preparation for deployments

#### Real-world tests.

Interim tests that reenact fieldwork, with a known number of samples present, help to check the abilities of each team. They can be conducted regularly and with increasing complexity in different environmental conditions, and should be standardised to the maximum possible.

#### Field work.

Volunteers should receive a solid theoretical and practical foundation before field deployments, including supervised transect searches. Training should also cover the assessment of site, habitat, and weather conditions to facilitate a successful transition to independent field work.

#### Target species.

The handlers need to be able to identify their target species and distinguish it from similar or related species. They should have basic ecological knowledge on their target species, such as seasonality, activity or movement patterns. This is necessary to confirm WDD alerts during field work.

#### Dog behaviour and condition.

Handlers need to learn reading the dog’s search behaviour, know and recognise the dog’s performance limits, understand the influence of weather and distractions on the search result, and be able to build up the dog’s motivation.

#### Maintenance training.

Even if dogs are properly trained, they should receive maintenance training on a regular basis to retain their performance level.

### Volunteer coordination

#### Coordination.

A dedicated coordination office should be set up to support volunteer WDD-teams, where regular contact to the participants can be guaranteed and, depending on the size of the group, participants can be actively consulted on the training/reporting situation.

#### Training.

Detection dog trainers and project coordinators should be available for the participants on a regular basis to ensure close supervision depending on the participant’s need. They offer theoretical knowledge adapted to the availability of the participants and enable multiple on-site offerings for practical sessions and real-world tests.

#### Feedback.

Regular feedback sessions and evaluations of both participants and dog trainers are necessary. In addition, feedback is often communicated in personal conversations, which should be initiated by the project coordinator. Feedback should be taken seriously, implemented if possible and communicated transparently.

#### Technical support.

Theoretical training and communication should be managed through a secure online platform that enables participants to access training materials, document progress, exchange information, and submit results. Participants should be provided with, or have access to, the necessary technical equipment, and dedicated IT support should be available to maintain the platform and resolve technical issues.

## Supporting information

S1 File Application of volunteers. (PDF)

S2 File Questionnaire during practical seminar. (PDF)

S3 Table Evaluation of motivation. (PDF)

S4 File Protocol of the field test. (PDF)

S5 Table Model selection results. (PDF)
