# Supplementary material for: Opportunities and challenges in training volunteer wildlife detection dog-handler teams for invasive plant monitoring
Source: PLoS One. 2026 Nov 4;21(11):e0359003. doi: 10.1371/journal.pone.0359003 (PMC13734480; doi:10.1371/journal.pone.0359003)
Supplement: Supplementary file 1 [file pone.0359003.s001.pdf]

S1 File. Application of volunteers

To open the applications for the individual cohorts, we created the following call: 'Sniffers wanted! Is your dog highly motivated by toys and food? Is he keen to work with you? Do you enjoy being out in nature and would you like to do more for nature conservation? Is time-consuming, systematic dog training just your thing? Then you could be the perfect IGAMon Dog team! We are [once again] looking for dog owners who would like to undergo training with us to become a detection dog for invasive plant species and then join us in monitoring their spread. Sounds exciting? Then apply now at [igamon.de] and join us on the plant hunt!' The call was distributed in social media and via email through the society Wildlife Detection Dogs e.V., as well as through word-of-mouth. A total of 21 and 34 people applied for the first and second cohort, respectively, through an online form. We asked for data about the handler and the dog, including previous experience with dog training in general and scent work specifically. Applicants also had to provide us with a video showing a snapshot of their training and skills. We opted for this hurdle because video analyses were planned during the training and the applicants needed to be familiar with the effort and technology involved.
