# Supplementary material for: Opportunities and challenges in training volunteer wildlife detection dog-handler teams for invasive plant monitoring
Source: PLoS One. 2026 Nov 4;21(11):e0359003. doi: 10.1371/journal.pone.0359003 (PMC13734480; doi:10.1371/journal.pone.0359003)
Supplement: Supplementary file 2 [file pone.0359003.s002.pdf]

SI2 File. Questionnaire during practical seminar

Questionnaire for dog owners

a. Team ID (Alternative: owner + dog)

b. Owner

i. Age

ii. Gender

iii. Experience in dog training before the start of the project

☐ very high ☐ high ☐ medium ☐ low

iv. Experience with search / detection dogs before the start of the project

☐ scent matching / object search ☐ tracking ☐ general scent work ☐ none

c. Dog

i. Age

ii. Breed

iii. intact/neutered

iv. Experience in dog training before the start of the project

☐ very high ☐ high ☐ medium ☐ low

v. Experience as a search / detection dog before the start of the project

☐ scent matching / object search ☐ tracking ☐ general scent work ☐ none

d. Training effort

i. Were you able to attend all the training webinars live?

☐ yes ☐ partly ☐ no

ii. Have you been able to watch missed training webinars afterwards?

☐ yes ☐ partly ☐ no ☐ not applicable

iii. Were you able to attend all the introductory webinars live?

☐ yes ☐ partly ☐ no

iv. Have you been able to watch missed introductory webinars afterwards?

☐ yes ☐ partly ☐ no ☐ not applicable

v. Please state your approximate number of training days per week

vi. What was the average length of a training session? How did this change over the training period?

vii. Was the training plan followed step by step? Or were steps skipped?

☐ exactly adhered to ☐ few steps skipped

☐ many steps skipped

At which point could steps be skipped?

viii. How often were single blind searches used/trained during training?

☐ (almost) every time ☐ once per training session

☐ every second training session ☐ rarely ☐ never

ix. How often were double blind searches used/trained during training?

☐ (almost) every time ☐ once per training session

☐ every second training session ☐ rarely ☐ never

x. What was the average approximate area of your previous search areas?

xi. What was the approximate area of your largest search area to date?

xii. What samples have you trained with so far?

☐ frozen ☐dried ☐sampled freshly ☐living/standing

xiii. Where did your training samples come from (if known)?

☐ UFZ (sent) ☐Bot. Garden (sent) ☐self-collected ☐other

xiv. How many different plant individuals were you able to train with?

☐1 ☐2-4 ☐5 and more

xv. Approximately how many parts did you cut each plant individual into?

☐1 ☐2-4 ☐5 and more

xv. Which parts of the plant were you able to use for training?

☐Leaves ☐Stem ☐Root ☐Flower

xiii. What type of reward was used in the training? What criteria were used to select it?

☐a toy ☐multiple different toys

☐a food type ☐different types of food

Criteria:
