# Supplementary material for: Opportunities and challenges in training volunteer wildlife detection dog-handler teams for invasive plant monitoring
Source: PLoS One. 2026 Nov 4;21(11):e0359003. doi: 10.1371/journal.pone.0359003 (PMC13734480; doi:10.1371/journal.pone.0359003)
Supplement: Supplementary file 3 [file pone.0359003.s003.pdf]

S3 Table. Evaluation of motivation

Definition of *Motivations* for volunteering selectable during the evaluation of the project by the volunteers. Participants could choose from a 6-point Likert scale, with higher scores indicating greater endorsement of those motivations. This design with no central neutral point aimed to reduce central tendency bias by preventing respondents from selecting a default 'neutral' response. A summary of the values chosen by participants is given (N = 23).

Motivation	Definition	Mean	Median	sd	Min	Max
Conservation	Doing something for a cause that is important to me; expressing my personal values; actively contributing to nature conservation	5.26	6	1.01	3	6
Responsibility	Helping to remedy shortcomings in nature conservation; fulfilling a socially meaningful task; initiating changes in nature conservation policy	4.52	5	1.53	1	6
Science	Support a scientific research project; engage in a professional exchange with scientists; better understand scientific processes; contribute to species detection and environmental monitoring; contribute to the exchange of knowledge between citizens and researchers	5.04	5	1.02	3	6
Community	Being part of a community that is committed to the same cause; getting involved with others; meeting people with similar interests	4.04	4	1.30	1	6
Qualification	Gain new perspectives on nature; learn new things through concrete action and apply them in practice; learn and apply theoretical expertise and methods	4.43	5	1.73	1	6
Self-efficacy	Making an important contribution; being needed; receiving recognition; realising oneself	3.52	4	1.38	1	6
Work-life balance	Recover from professional demands by spending time in the great outdoors; work in a self-determined way; do something meaningful in your free time	3.65	4	1.85	1	6
Career	Gain experience that can also be useful in your job; expand your professional skills; make and maintain contacts that can be beneficial for your professional development	3.61	4	1.90	1	6
Dog training	Learn more about working and training with dogs; strengthen the bond with your own dog; learn new things together with your own dog	5.96	6	0.21	5	6
