# Supplementary material for: Opportunities and challenges in training volunteer wildlife detection dog-handler teams for invasive plant monitoring
Source: PLoS One. 2026 Nov 4;21(11):e0359003. doi: 10.1371/journal.pone.0359003 (PMC13734480; doi:10.1371/journal.pone.0359003)
Supplement: Supplementary file 4 [file pone.0359003.s004.pdf]

SIV: Protocol of the field test

Name	Dog	Breed	Federal State	Target Species	Team ID	Date

Preparation:		Video:
Time:	Leash:	
Temperature:	Humidity:	Weather:
Wind:	Wind direction:	Search Area:

Search 1 (25 x 25 m / 200 m Transect):
Number of samples: Sample origin:

Grid (25 cells, á 5 x 5 m)

Sample	Grid	Size / Age / Plant part	found by dog	found by handler	Time [min]	Alert	Distractions	VideoNr
Sample 1								
Sample 2								
Sample 3								
Sample 4								
Sample 5								

Optional:

Search 2 (x   m /   m Transect):
Number of samples: Sample origin: wild plants, not touched before

Sample	Grid	Size/Age/Plant part	found by dog	found by handler	Time [min]	Alert	Distractions	VideoNr
Sample 1								
Sample 2								
Sample 3								
Sample 4								
Sample 5								
