# Supplementary material for: Opportunities and challenges in training volunteer wildlife detection dog-handler teams for invasive plant monitoring
Source: PLoS One. 2026 Nov 4;21(11):e0359003. doi: 10.1371/journal.pone.0359003 (PMC13734480; doi:10.1371/journal.pone.0359003)
Supplement: Supplementary file 5 [file pone.0359003.s005.pdf]

S5 Table. Model selection results

Model comparison for all model combinations within ΔAIC < 2. Models refer to the question and the hypothesis according to Table 3 (main text). Parameter definitions can be found in Table 2 (main text). df - degrees of freedom; logLik - log Likelihood

**Model**	**Predictor**	**Df**	**LogLik**	**AIC**	**ΔAIC**
**Q1 - Team**	Team	27	-167.79	389.6	0
**Q1 - Basics**	Dog age + Chosen alert behaviour + Team experience + FCI + Cohort + Reward type	17	-194.58	432.2	0
	Chosen alert behaviour + Team experience + FCI + Cohort + Reward type	16	-196.18	424.4	1.19
**Q1 - Training**	Distraction + Target species	5	-247.91	505.8	0
	Distraction + Training step + Target species	6	-247.28	506.6	0.73
	Distraction + Target species + Sample odour	6	-247.90	507.8	1.97
**Q2 - Team**	1	1	-73.77	149.5	0
	Team	26	-49.62	151.2	1.7
**Q2 - Basics**	Chosen alert behaviour + Team experience + FCI + Reward type	14	-53.61	135.2	0
	Dog age + Chosen alert behaviour + FCI + Reward type	12	-55.68	135.4	0.14
	Chosen alert behaviour + FCI + Reward type	11	-56.78	135.6	0.33
	Dog age + Chosen alert behaviour + Team experience + FCI + Reward type	15	-53.10	136.2	0.98
	FCI + Reward type	9	-59.24	136.5	1.26
	Chosen alert behaviour + Team experience + FCI + Cohort + Reward type	15	-53.57	137.1	1.92
**Q2 - Training**	De.ave.duration + Training step + Distraction + Target species	6	-61.91	135.8	0
	De.ave.duration + Distraction + Target species	5	-62.96	135.9	0.12
	De.ave.duration + Distraction + Target species + Sample odour	6	-62.46	136.9	1.10
	De.ave.duration + Training step + Distraction + Target species + Sample odour	7	-61.70	137.4	1.59
**Q3 - On-site conditions**	Sample condition + Sample length + Target species + Cohort + Sample condition:Sample length	7	-43.23	100.46	0
	Sample length + Target species + Cohort	5	-45.42	100.84	0.38
	Sample condition + Sample length + Cohort + Sample condition:Sample length	5	-46.08	102.16	1.70
	Sample length + Target species + Wind + Cohort	7	-44.14	102.29	1.83
**Q3 - Training conditions**	Plant_parts + Steps_skipped	5	-57.47	124.93	0.00
	Plant_parts + Steps_skipped + Fresh_training	6	-57.25	126.50	1.57
	Plant_parts + Steps_skipped + Dried	6	-57.27	126.54	1.61
	Plant_parts + Team experience	4	-59.32	126.63	1.70

	Plant_parts + Steps_skipped + Team experience	6	-57.36	126.72	1.79
	Plant_parts	3	-60.44	126.88	1.94
	Plant_parts + Steps_skipped + log(Search area)	6	-57.46	126.93	1.99
	Plant_parts + Steps_skipped + Training days/week	6	-57.46	126.93	1.99
**Q4 - On-site conditions**	Wind + Cohort	5	-39.44	88.88	0
	Wind + Cohort + Fresh_test	6	-38.78	89.55	0.68
	Wind + Cohort + Rel_goal	6	-39.32	90.63	1.75
	Wind + Cohort + Diff_goal	6	-39.42	90.85	1.97
**Q4 - Training conditions 1**	Plant_parts	4	-51.04	110.08	0.00
	Plant_parts + log(Search_area)	5	-50.29	110.59	0.51
	Plant_parts + Dried	5	-50.77	111.54	1.47
	Plant_parts + Fresh_training	5	-50.94	111.88	1.80
**Q4 - Training conditions 2**	Session_duration + Steps_skipped	5	-49.63	109.27	0.00
	Session_duration + Steps_skipped + Team experience	6	-49.11	110.21	0.95
	Session_duration + Steps_skipped + Training_days/week	5	-49.43	110.86	1.59
**Q5 – Team performance**	1	1	-15.16	32.3	0
	Cohort	2	-14.79	33.6	1.26
	Diff_goal	2	-14.87	33.7	1.42
	Team score	2	-15.03	34.1	1.75
	Rel_goal	2	-15.09	34.2	1.86
**Q5 – Motivation**	Community + Conservation + Science	4	-8.23	24.5	0
	Community + Conservation + Science + Self-efficiency	5	-7.25	24.5	0.05
	Science	2	-10.44	24.9	0.41
	Community + Conservation + Science + Self-efficiency + Career	6	-6.44	24.9	0.42
	Conservation + Science	3	-9.72	25.4	0.97
	Community + Science	3	-9.74	25.5	1.01
	Career + Science	3	-9.78	25.5	1.09
	Community + Conservation + Qualification + Science	5	-7.82	25.6	1.18
	Community + Conservation + Qualification + Science + Self-efficiency	6	-6.84	25.7	1.22
	Community + Conservation + Science + Career	5	-7.95	25.9	1.44
	Community + Qualification + Science	4	-9.02	26.0	1.58
	Qualification + Science	3	-10.12	26.2	1.79
	Conservation + Science + Career	4	-9.19	26.4	1.92
	Community + Science + Career	4	-9.19	26.4	1.92

5
